# Effects of age and time since injury on traumatic brain injury blood biomarkers: a TRACK-TBI study

**DOI:** 10.1093/braincomms/fcac316

**Published:** 2022-12-01

**Authors:** Raquel C Gardner, Ava M Puccio, Frederick K Korley, Kevin K W Wang, Ramon Diaz-Arrastia, David O Okonkwo, Ross C Puffer, Esther L Yuh, John K Yue, Xiaoying Sun, Sabrina R Taylor, Pratik Mukherjee, Sonia Jain, Geoffrey T Manley, the TRACK-TBI Investigators, Venkata R Feeser, Adam R Ferguson, Etienne Gaudette, Shankar Gopinath, C Dirk Keene, Christopher Madden, Alastair Martin, Michael McCrea, Randall Merchant, Pratik Mukherjee, Laura B Ngwenya, Claudia Robertson, Nancy Temkin, Mary Vassar, John K Yue, Ross Zafonte

**Affiliations:** Department of Neurology, University of California, San Francisco, San Francisco, CA 94143, USA; Neurology Division, San Francisco Veterans Affairs Medical Center, San Francisco, CA 94121, USA; Sheba Medical Center, Ramat Gan, 52621, Israel; Department of Neurological Surgery, University of Pittsburgh Medical Center, Pittsburgh, PA 15213, USA; Department of Emergency Medicine, University of Michigan, Ann Arbor, MI 48109, USA; Departments of Emergency Medicine, Psychiatry, and Neuroscience, McKnight Brain Institute, University of Florida, Gainesville, FL 32610, USA; Brain Rehabilitation Research Center (BRRC), Malcom Randall VA Medical Center, North Florida/South Georgia Veterans Health System, 1601 SW Archer Rd., 32608, USA; Department of Neurology, University of Pennsylvania, Philadelphia, PA 19104, USA; Department of Neurological Surgery, University of Pittsburgh Medical Center, Pittsburgh, PA 15213, USA; Department of Neurological Surgery, University of Pittsburgh Medical Center, Pittsburgh, PA 15213, USA; Department of Neurological Surgery, Mayo Clinic, Rochester, MN 55901, USA; Department of Radiology, University of California, San Francisco, San Francisco, CA 94143, USA; Department of Neurological Surgery, University of California, San Francisco, San Francisco, CA 94143, USA; Herbert Wertheim School of Public Health and Human Longevity Science, University of California San Diego, San Diego, CA 92161, USA; Department of Neurological Surgery, University of California, San Francisco, San Francisco, CA 94143, USA; Department of Radiology, University of California, San Francisco, San Francisco, CA 94143, USA; Herbert Wertheim School of Public Health and Human Longevity Science, University of California San Diego, San Diego, CA 92161, USA; Department of Neurological Surgery, University of California, San Francisco, San Francisco, CA 94143, USA

**Keywords:** traumatic brain injury, aging, head CT, biomarkers, diagnostic

## Abstract

Older adults have the highest incidence of traumatic brain injury globally. Accurate blood-based biomarkers are needed to assist with diagnosis of patients across the spectrum of age and time post-injury. Several reports have suggested lower accuracy for blood-based biomarkers in older adults, and there is a paucity of data beyond day-1 post-injury. Our aims were to investigate age-related differences in diagnostic accuracy and 2-week evolution of four leading candidate blood-based traumatic brain injury biomarkers—plasma glial fibrillary acidic protein, ubiquitin carboxy-terminal hydrolase L1, S100 calcium binding protein B and neuron-specific enolase—among participants in the 18-site prospective cohort study Transforming Research And Clinical Knowledge in Traumatic Brain Injury. Day-1 biomarker data were available for 2602 participants including 2151 patients with traumatic brain injury, 242 orthopedic trauma controls and 209 healthy controls. Participants were stratified into 3 age categories (young: 17–39 years, middle-aged: 40–64 years, older: 65–90 years). We investigated age-stratified biomarker levels and biomarker discriminative abilities across three diagnostic groups: head CT-positive/negative; traumatic brain injury/orthopedic controls; and traumatic brain injury/healthy controls. The difference in day-1 glial fibrillary acidic protein, ubiquitin carboxy-terminal hydrolase L1 and neuron-specific enolase levels across most diagnostic groups was significantly smaller for older versus younger adults, resulting in a narrower range within which a traumatic brain injury diagnosis may be discriminated in older adults. Despite this, day-1 glial fibrillary acidic protein had good to excellent performance across all age-categories for discriminating all three diagnostic groups (area under the curve 0.84–0.96; lower limit of 95% confidence intervals all >0.78). Day-1 S100 calcium-binding protein B and ubiquitin carboxy-terminal hydrolase L1 showed good discrimination of CT-positive versus negative only among adults under age 40 years within 6 hours of injury. Longitudinal blood-based biomarker data were available for 522 hospitalized patients with traumatic brain injury and 24 hospitalized orthopaedic controls. Glial fibrillary acidic protein levels maintained good to excellent discrimination across diagnostic groups until day 3 post-injury irrespective of age, until day 5 post-injury among middle-aged or younger patients and until week 2 post-injury among young patients only. In conclusion, the blood-based glial fibrillary acidic protein assay tested here has good to excellent performance across all age-categories for discriminating key traumatic brain injury diagnostic groups to at least 3 days post-injury in this trauma centre cohort. The addition of a blood-based diagnostic to the evaluation of traumatic brain injury, including geriatric traumatic brain injury, has potential to streamline diagnosis.

## Teaser

Gardner *et al.* report that blood-based glial fibrillary acidic protein level has good-excellent discrimination across key traumatic brain injury diagnostic groups until day 3 post-injury irrespective of age, until day 5 post-injury among middle-aged or younger patients and until week 2 post-injury among young patients.

## Graphical Abstract

**Graphical abstract:**
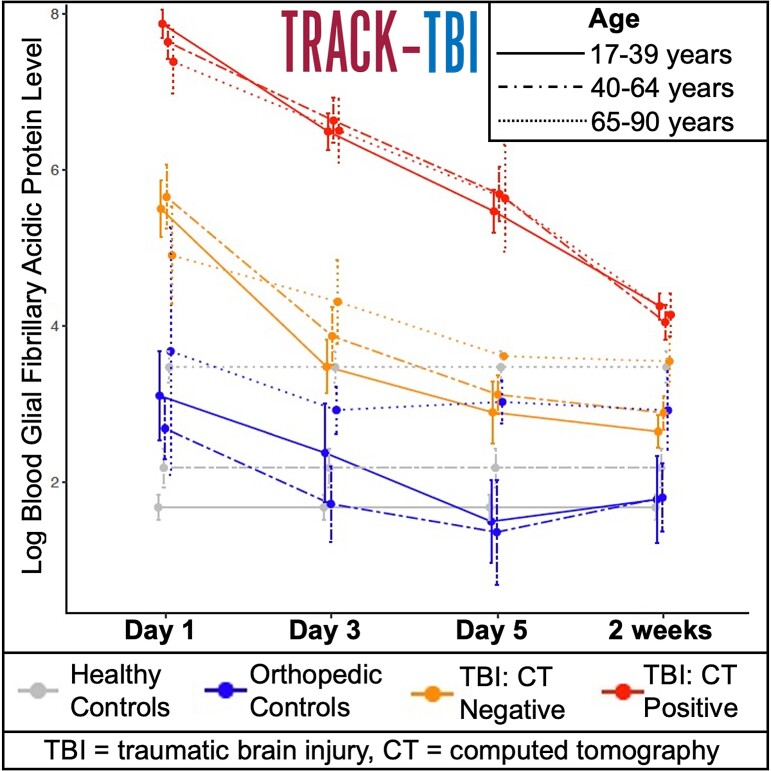

## Introduction

Traumatic brain injury (TBI) affects tens of millions of individuals worldwide each year and is recognized as a global health priority with substantial associated morbidity, mortality and cost.^1^ Incidence of TBI is on the rise, particularly among the elderly who now have the highest and fastest rising incidence of TBI in the USA and globally mostly due to ground-level falls.^1,2^ In the USA alone, more than 1 in 50 adults age 75 years and older sustain a TBI each year.^3,4^ A major limitation in clinical care and research of patients with TBI is that initial diagnosis relies on clinical presentation: that is, a reported history of trauma, often vague neurological symptoms (e.g. alteration or loss of consciousness or amnesia), and a rudimentary neurological examination [e.g. the Glasgow Coma Scale (GCS)]. Based on this clinical assessment, a decision is made about whether a head CT is indicated. This approach presents a challenge, particularly for patients who are unable to provide a history or in whom non-focal neurological symptoms may be attributed to something other than TBI (i.e. intoxication, cardiogenic syncope, polytrauma with hypovolemic shock). As a result, many unnecessary head CTs are obtained.

Diagnostic challenges are magnified in the elderly population in whom pre-existing conditions frequently result in complex and often unwitnessed combinations of falls, head trauma, syncope and delirium, and in whom large intracranial haemorrhages may expand silently within the atrophied cerebral hemispheres without producing clinical signs or symptoms.^5^ It is therefore not surprising that older patients suffer disproportionate rates of misdiagnosis, erroneous pre-hospital triage and delays in definitive assessment and management compared with younger patients.^6^ As population aging continues, there is a clear need for accurate, low-cost, minimally invasive diagnostic tests for acute TBI with demonstrated accuracy in both younger and older individuals.

There has been considerable recent progress in development of blood-based proteomic biomarkers for acute TBI diagnosis.^7,8^ Currently, there are several candidate blood-based biomarkers that are at varying levels of development, validation and clinical implementation for the diagnosis of acute TBI. Among the leading candidates are glial fibrillary acidic protein (GFAP), ubiquitin carboxy-terminal hydrolase L1 (UCH-L1), S100 calcium binding protein B (S100B) and neuron-specific enolase (NSE).^9–14^ S100B biomarker is part of the Scandinavian neurotrauma head CT guidelines.^15^ GFAP and UCH-L1 assays were recently approved by the US Food and Drug Administration (FDA) to aid in the diagnosis of mild TBI amongst patients presenting within 12 hours of injury^16^ and subsequently received further FDA approval to be measured on the Abbott I-STAT handheld device that returns results within 15 minutes.^17^

Most prior studies of blood-based TBI biomarkers have not specifically evaluated accuracy in older adults.^18^ Among those that have specifically investigated accuracy in older adults, all have focused on the hyper-acute or acute phase in patients presenting within 3,^19,20^ 6,^21^ 12^22^ or 24 hours post-injury.^23^ Overall, these studies found that while sensitivity of plasma S100B, GFAP, and UCH-L1 for identification of CT-evidence of intracranial trauma was usually good, specificity was significantly reduced in older versus younger patients, suggesting that while these assays may improve appropriate triage and reduce delays in management, they may not substantially reduce the need for head CTs in older patients due to the expected high rate of false positives. The effect of advanced age on performance of NSE has not been studied. Furthermore, older adults are significantly more likely than younger patients to present in a delayed fashion (e.g. > 24 hours) post-injury.^24^ Additionally, the blood-based biomarker assay technology has improved over time, and many are far more sensitive, with much lower limits of detection/quantification, than older assays used in prior studies.^23,25,26^ Thus, further investigation of the impact of age and time since injury on evolution and performance of leading blood-based biomarker candidates is critically important to guide appropriate use and warn against potential misuse.

The aims of this study were to investigate age-related differences in diagnostic accuracy and 2-week evolution of four of the leading blood-based TBI biomarkers—plasma S100B, GFAP, UCH-L1, and NSE—in a very large cohort of *N* = 2602 patients presenting within 24 hours of all-severity TBI as part of the 18-site prospective cohort study Transforming Research andClinical Knowledge in TBI (TRACK-TBI). Specifically, we investigated the age-related accuracy of these 4 TBI diagnostic blood-based biomarkers for: (i) distinguishing head CT-positive versus CT-negative participants with TBI, (ii) distinguishing participants with TBI from those with orthopedic injury, and (iii) distinguishing participants with TBI from healthy non-injured controls. We additionally investigated the effect of time since injury including detailed analysis of 2-week blood-based biomarker trajectories by age and CT findings in the subset of hospitalized patients who underwent serial blood sampling. Lastly, we had the unique opportunity to explore independent effects of age on blood-based TBI biomarkers via head-to-head comparison of cross-sectional biomarker elevations across age-categories among healthy and orthopedic controls relative to individuals with TBI.

## Materials and methods

### Study population

Participants with TBI, orthopedic trauma controls (OCs), and healthy non-injured controls (HCs) age 17 years and older were enrolled in the prospective TRACK-TBI study (ClinicalTrials.gov: NCT02119182) from 26 February 2014 to 25 September 2019 as described previously.^26–29^ TRACK-TBI is a prospective cohort study that enrolled patients with TBI who presented to the emergency department of one of 18 participating Level 1 trauma centres within 24 hours of TBI and received clinical evaluation with head CT by the emergency department clinician, based on practice guidelines.^30^ Exclusion criteria included: significant polytrauma that would interfere with follow-up, prisoners or patients in custody, pregnancy, patients on psychiatric hold, major pre-existing medical, psychiatric, or neurological disease that would interfere with follow-up or outcome assessment (e.g. schizophrenia, dementia, terminal cancer), low likelihood of follow-up, participation in an interventional trial, penetrating TBI or spinal cord injury with American Spinal Injury Association grade C or worse.

Baseline clinical data were collected, including demographics, medical history, and injury characteristics including GCS, trauma mechanism, loss of consciousness, post-traumatic amnesia and initial hospital or emergency department course. OCs were enrolled using similar procedures except that they were required to present with isolated trauma to their limbs, pelvis and/or ribs and had an Abbreviated Injury Score less than four for those body regions. Patients were excluded from enrolment as OCs if they had loss or alteration of consciousness, post-traumatic amnesia or any clinical findings suggestive of head injury. HCs were recruited from friends or relatives of TRACK-TBI participants (‘friend’ HCs) or through public advertisements at participating sites (‘community’ HCs). HCs were eligible for inclusion if they had no history of TBI, concussion or any traumatic bodily injury in the 12 months prior to enrolment. ‘Friend’ HCs completed the same protocol of assessments as participants with TBI including a comprehensive baseline interview that assessed past medical and psychiatric history; ‘community’ HCs completed limited baseline assessments that did not include past medical or psychiatric history.

All OCs and HCs provided written informed consent. Participants with TBI either provided written informed consent or, if they lacked capacity, consent was obtained from their legal representative. Occasionally, blood was drawn initially under emergency waiver of consent. In these cases, in-person consent from the patient or their legal representative was obtained at the earliest possible opportunity and no more than 72 hours post-injury. For minors aged 17, consent was obtained from patients and/or their legal guardian.

### Blood collection and analysis

Blood was drawn via intravenous phlebotomy or directly from an arterial catheter on day 1 (within 24 hours) post-injury for participants with TBI and OCs. Blood was drawn via intravenous phlebotomy for HCs at the time of enrolment. In a subset of hospitalized patients with TBI and OC participants, blood collection was repeated on day 3, day 5, and week 2 post-injury. All samples were dated and time stamped.

Samples were processed and stored according to the National Institute of Neurological Disorders and Stroke Common Data Elements Biospecimens and Biomarkers Working Group consensus recommendations for plasma preparation.^31^ Plasma aliquots of 500 microliters were prepared for each subject and frozen at minus 80°C for future batch processing. Samples were batch-shipped in temperature-controlled overnight express freight containers to the TRACK-TBI Biospecimens Repository at the University of Pittsburgh Medical Center (Pittsburgh, PA, USA). These samples were part of the ‘TRACK-TBI Biomarker Cohort,’ which included baseline blood samples for *N* = 2151 patients with TBI, *N* = 242 OCs and *N* = 209 HCs.

Details of sample analysis for each assay were previously described in detail.^26^ Briefly, GFAP and UCH-L1 were measured on two platforms: (i) A prototype point-of-care i-STAT™ Alinity™ System (*N* = 1429) which uses a sandwich enzyme-linked immunosorbent assay (ELISA) method with electrochemical detection of the resulting enzyme signal and (ii) a prototype core lab Abbott ARCHITECT® platform (*N* = 964) which uses two-step sandwich assays that use a chemiluminescent microparticle immunoassay technology. Test time for the i-STAT™ Alinity™ System is approximately 15 minutes per assay. ARCHITECT® values were converted to iSTAT equivalents using two previously derived equations: iSTAT = −12.36 + 1.02*ARCHITECT for GFAP (Spearman’s correlation coefficient = 0.985) and iSTAT = −3.29 + 0.72*ARCHITECT for UCH-L1 (Spearman’s correlation coefficient = 0.933).^25^ S100B and NSE were measured using electrochemiluminescence immunoassay (ECLIA) on the Roche Elecsys System. Reportable range (RR), limit of detection (LoD), limit of quantification (LoQ) and coefficient of variation (CV) for each assay are as follows: GFAP iSTAT RR 15 to 50 000 pg/ml, LoD 15 pg/ml, LoQ 25 pg/ml, CV 2.8 to 14.2%; GFAP ARCHITECT® RR 2–50 000 pg/ml, LoD 2 pg/ml, LoQ 5 pg/ml, CV 2.0–5.6%; UHCL1 iSTAT RR 10 to 20 000 pg/ml, LoD 10 pg/ml, LoQ 20 pg/ml, CV 5.0 to 10.0%; UCHL1 ARCHITECT® RR 10–25 000 pg/ml, LoD 10 pg/ml, LoQ 20 pg/ml, CV 2.0–5.7%; S100B RR 0.005–39 µg/L, LoD 0.005 µg/L, CV 20%; NSE RR 0.05 to 370 ng/ml, LoD 0.05 ng/ml, CV 20%.

### Head CT imaging

The first head CT obtained for each patient at the time of presentation with TBI was deidentified, uploaded to a central imaging database at the Laboratory of NeuroImaging (University of Southern California, Los Angeles, CA, U.S.), and independently evaluated by a board-certified neuroradiologist according to the NINDS Common Data Element Neuroimaging Working Group consensus recommendations.^32^ The neuroradiologist was blinded to all clinical information except age and TBI diagnosis. CT scans were read as positive (CT+) if there was any evidence of acute intracranial traumatic pathology consistent with TBI (e.g. brain contusion, subarachnoid haemorrhage, subdural haematoma, epidural haematoma, intraventricular haemorrhage). CT scans were read as negative (CT−) if there was no evidence of acute intracranial traumatic pathology consistent with TBI.

### Statistical analysis

Participants were categorized as young (age <40 years), middle-aged (age 40–64 years), or older (age ≥65 years). Baseline demographics, pre-existing conditions, TBI characteristics, CT positivity, and time from injury to blood draw were compared across age categories using Wilcoxon rank sum test for continuous variables and Fisher’s exact test for categorical variables.

Diagnostic performance of blood-based biomarkers was investigated for the following diagnostic groups: participants with TBI identified as CT + versus CT−, participants with TBI versus OCs, participants with TBI versus HCs. Day-1 biomarker levels were summarized and compared by diagnostic group, stratified by sampling time interval (0–6, 7–12, 13–24 hours post-injury), and age category. Comparisons were made using Wilcoxon Rank Sum test. Linear regression models were also conducted with log-transformed biomarker levels as the outcome, diagnostic group, age category and interaction between the two as predictors to assess whether group difference in biomarker levels (e.g. difference in logGFAP between CT + and CT− groups) differed by age category. Receiver operating characteristic (ROC) analysis was conducted to compare the performance of these biomarkers among the different age groups in predicting: (i) CT + versus CT−; (ii) TBI versus OC; and (iii) TBI versus HC. ROC curves were plotted and area under the curve (AUCs) were calculated with 95% confidence intervals (CIs). AUCs of < 0.7 were considered poor, 0.7–0.8 fair, 0.8–0.9 good and 0.9–1.0 excellent. Delong’s test was used for AUC comparisons. Because the FDA has recently cleared diagnostic cut-offs for the iSTAT GFAP and iSTAT UCHL1 assays for discriminating CT-positivity^33^ and Roche has proposed a diagnostic cut-off for the Elecsys S100b assay for discriminating CT-positivity,^34^ we conducted a cut-off analysis to estimate sensitivity and specificity of these established diagnostic thresholds, stratified by age and sampling time, for discriminating CT + versus CT− on day 1.

Longitudinal blood-based biomarker data (day1, day 3, day 5 and week 2) were summarized and plotted by diagnostic group over time, stratified by the three age groups. AUCs were calculated to determine the diagnostic accuracy of blood-based biomarkers over multiple days.

Analyses were conducted using statistical software R (version 4.1.2; http://www.r-project.org). *P*-values < 0.05 were considered statistically significant.

## Results

### Cohort characteristics

Day-1 blood-based biomarker data were available for 2602 participants, including 2151 participants with TBI, 242 OCs and 209 HCs. Head CT data were available for all 2151 of these TBI participants. Longitudinal blood-based biomarker data were available for 522 hospitalized participants with TBI and 24 hospitalized OCs who had blood drawn on day 1, week 2 and at least one measure from day 3 or day 5. Head CT data were available for all 522 of these TBI participants (see Supplemental Figure 1 for participant flow diagram). Baseline demographic and clinical characteristics of participants with TBI stratified by age category are shown in Table 1. Compared with younger and middle-aged patients, older patients had a significantly lower proportion of Black or Hispanic race, higher level of education, higher prevalence of nearly all pre-existing medical conditions, but lower prevalence of alcohol abuse and illicit drug use. While there were no significant age-related differences in presenting GCS and the vast majority of TBIs were GCS 13–15, there was a high proportion of CT-positivity and ICU admission that increased with increasing age category. Mean times from TBI to blood draw across age groups ranged from 14.3–16.0 and was significantly longer in the oldest age group. Baseline characteristics of OCs, ‘friend’ HCs and ‘community’ HCs are shown in Supplemental Tables 1–3.

**Table 1:** Characteristics of patients with traumatic brain injury stratified by age

Characteristic *N*(%) or mean ± standard deviation	17–39 years *N* = 1153	40–64 years *N* = 758	65–90 years *N* = 240	*P*-value
Age, years	27.0 ± 6.1	51.6 ± 6.9	72.5 ± 6.4	<0.001
Female	332 (28.79%)	240 (31.66%)	83 (34.58%)	0.138
Race				
White	855 (74.87%)	594 (79.41%)	204 (86.08%)	<0.001
Black	202 (17.69%)	119 (15.91%)	20 (8.44%)	
Other	85 (7.44%)	35 (4.68%)	13 (5.49%)	
Hispanic	265 (23.23%)	127 (16.96%)	16 (6.78%)	*<*0.001
Education, years	13.1 ± 2.4	13.6 ± 3.2	14.2 ± 3.2	<0.001
Past medical history				
Hypertension	31 (2.69%)	186 (25.54%)	123 (51.25%)	<0.001
Hyperlipidaemia	9 (0.78%)	63 (8.31%)	53 (22.08%)	<0.001
Ischaemic heart disease	0 (0%)	5 (0.66%)	12 (5.00%)	*<*0.001
Stroke or transient ischaemic attack	3 (0.26%)	9 (1.19%)	17 (7.08%)	*<*0.001
Diabetes	15 (1.3%)	94 (12.4%)	59 (24.58%)	*<*0.001
Renal disease	43 (3.73%)	55 (7.26%)	25 (10.42%)	*<*0.001
Pulmonary disease	126 (10.94%)	110 (14.51%)	47 (19.58%)	0.001
Prior traumatic brain injury	351 (31.91%)	215 (30.11%)	56 (25.0%)	0.131
Psychiatric history	244 (21.18%)	195 (25.73%)	57 (23.75%)	0.072
Alcohol abuse	514 (48.26%)	277 (39.57%)	60 (27.40%)	<0.001
Illicit drug use	404 (38.04%)	105 (15.15%)	8 (3.72%)	<0.001
Injury mechanism				
Fall	172 (14.94%)	240 (31.96%)	139 (57.92%)	<0.001
Road traffic accident	781 (67.85%)	392 (52.2%)	83 (34.58%)	
Violence/assault	90 (7.82%)	54 (7.19%)	5 (2.08%)	
Other/unknown	108 (9.38%)	65 (8.66%)	13 (5.42%)	
Presenting Glasgow Coma Scale				
3–8	158 (14.04%)	104 (14.04%)	22 (9.44%)	0.182
9–12	56 (4.98%)	39 (5.26%)	7 (3%)	
13–15	911 (81.00%)	598 (80.7%)	204 (87.55%)	
Disposition				
Emergency department discharge	270 (23.42%)	161 (21.24%)	37 (15.42%)	0.003
Hospital ward admit	415 (35.99%)	243 (32.06%)	77 (32.08%)	
Intensive care unit admit	468 (40.59%)	354 (46.7%)	126 (52.5%)	
Loss of consciousness				
Yes	1002 (87.13%)	637 (84.15%)	182 (75.83%)	<0.001
No	101 (8.78%)	77 (10.17%)	46 (19.17%)	
Unknown	47 (4.09%)	43 (5.68%)	12 (5.00%)	
Post-traumatic amnesia				
Yes	836 (72.7%)	537 (70.94%)	165 (68.75%)	0.678
No	180 (15.65%)	123 (16.25%)	45 (18.75%)	
Unknown	134 (11.65%)	97 (12.81%)	30 (12.5%)	
Intracranial trauma on CT	461 (39.98%)	391 (51.58%)	171 (71.25%)	<0.001
Subarachnoid haemorrhage	315 (27.37%)	297 (39.39%)	128 (53.33%)	<0.001
Epidural haematoma	114 (9.9%)	47 (6.24%)	7 (2.92%)	<0.001
Subdural haematoma	245 (21.29%)	223 (29.61%)	108 (45.0%)	<0.001
Intraventricular haemorrhage	41 (3.56%)	34 (4.51%)	26 (10.83%)	<0.001
Contusion	228 (19.81%)	161 (21.38%)	56 (23.33%)	0.421
Blood draw time post-injury, hours	14.3 ± 6.3	14.7 ± 6.6	16.0 ± 6.7	<0.001

### Day 1 biomarker cohort results

**Figure 1:**
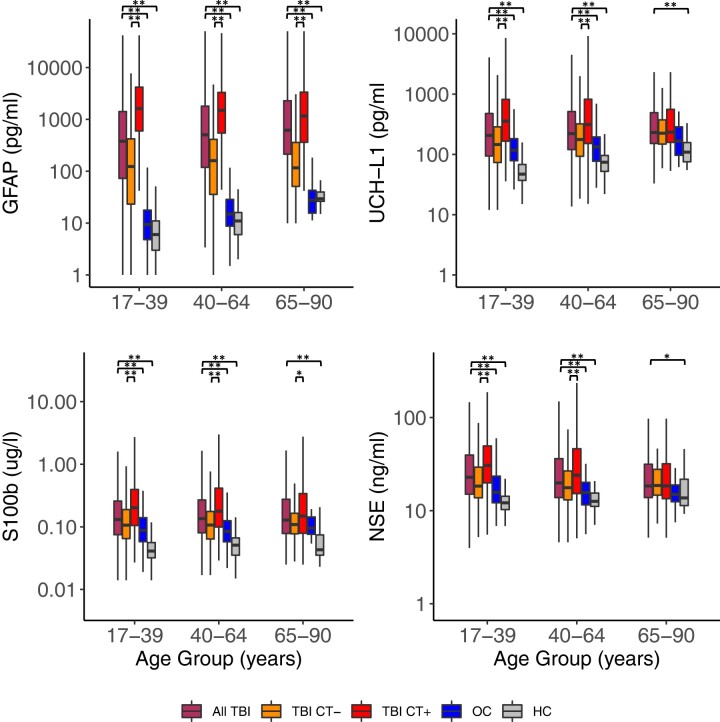
**Day 1 log-transformed blood-based biomarker levels stratified by diagnostic group and age.** Boxplots of day 1 blood-based biomarker levels (in log10 scale) are shown for GFAP, UCH-L1, S100b and NSE stratified by age group and then by diagnostic group [all TBI; TBI with a negative head CT (TBI CT−); TBI with a positive head CT (TBI CT+); orthopedic control (OC); healthy control (HC)]. The lower and upper ends of each box represent the 25th and 75th percentile; the line going through each box represents the median value; upper whisker indicates the smaller value of: the maximum value or 75th percentile +1.5 * IQR, and lower whisker indicates the larger value of: the minimum value or.25th percentile −1.5*IQR. Y-axis is marked in actual concentrations to facilitate clinical interpretation. Specific blood-biomarker levels and results of Wilcoxon Rank Sum tests comparing levels across diagnostic groups, stratified by age, are reported in Table 2. Statistically significant pairwise comparisons are indicated with brackets and **(indicating *P* < 0.001) or *(indicating *P* < 0.05).

**Figure 2:**
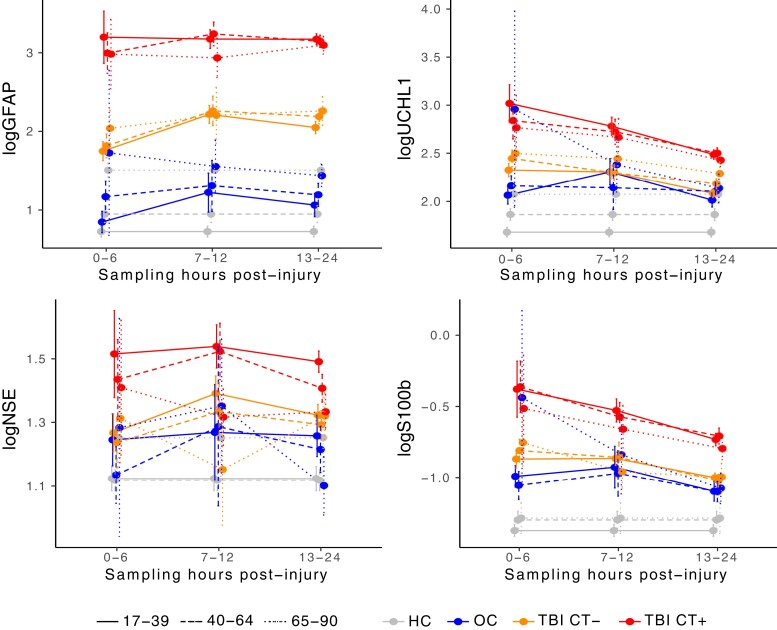
**Day 1 blood-based biomarkers by diagnostic group, sampling time intervals, and age-category.** Mean and 95% CIs are plotted for log-transformed blood-biomarker levels for GFAP, UCHL-1, NSE, and S100b. Each participant contributes only a single measurement and these levels are cross-sectional, not longitudinal. They are plotted to inform the optimal blood sampling time for each age group. Optimal timing of GFAP sampling is likely 7–24 hours as both CT + and CT− TBI remain fairly stably elevated across all age groups during this sampling interval. Optimal timing of UCHL1 and S100b sampling is likely 0–6 as levels generally begin to fall among CT + and CT− TBI by 7 + hours post-injury. NSE levels are less predictable with levels among CT + and CT− falling after 0–6 hours among elderly but rising up to 12 hours post-injury among middle-aged and younger adults. This figure also highlights the handful of extreme blood-biomarkers elevations among elderly OCs at 0–6 hours post-injury across all measured biomarkers. Specific blood-biomarker levels and results of Wilcoxon rank sum tests comparing levels across diagnostic groups, stratified by age and sampling time intervals, are reported in Supplemental Table 4.

Median and interquartile range (IQR) for each blood-based biomarker level by diagnostic group and age category are shown in Table 2, and log-transformed values are plotted in Fig. 1. Median and IQR for each blood-based biomarker level by diagnostic and age category are shown stratified by hours post-injury (0–6, 7–12, 13–24 hours) in Supplemental Table 4 and log-transformed values (mean with 95% CI) are plotted in Fig. 2. Interestingly, GFAP levels are maximal in CT + TBI immediately at 0–6 hours post-TBI, in CT− TBI at 7–12 hours, and then are stably elevated throughout day 1. UCHL1 and S100b are maximal at 0–6 hours post-TBI and decline thereafter. Elderly OCs have very high elevations in all biomarkers at 0–6 hours but these fall rapidly for GFAP, UCHL1, and S100b. Fig. 2 clearly illustrates the age-effect on basal levels of each biomarker among HCs with GFAP being the most impacted by age-related elevation in elderly HCs, followed by UCHL1. S100b is only minimally higher in middle-aged and older versus younger HCs while NSE does not appear to be impacted at all by age among HCs.

**Table 2:** Day 1 blood-based biomarker levels stratified by diagnostic group and age

Values are median (interquartile range)	17–39 years	*P*-value	40–64 years	*P*-value	65–90 years	*P*-value
GFAP, pg/mL						
TBI/CT+	1612.7 (596.4–4183.3)		1497.2 (544.8–3301.0)		1162.0 (360.8–3351.3)	
TBI/CT− (Ref: TBI/CT+)	122.4 (22.3–420.3)	<0.001	159.0 (34.7–411.2)	<0.001	115.2 (50.0–357.7)	<0.001
All TBI	378.0 (71.7–1414.2)		503.4 (117.8–1801.8)		618.8 (212.3–2291.5)	
OC (Ref: All TBI)	8.4 (3.8–16.8)	<0.001	14.0 (7.7–27.6)	<0.001	26.6 (14.6–41.9)	<0.001
HC (Ref: All TBI)	5.0 (2.0–10.0)	<0.001	10.0 (5.0–15.0)	<0.001	28.5 (25.3–38.8)	<0.001
UCH-L1, pg/mL						
TBI/CT+	356.0 (165.8–817.0)		313.4 (151.5–821.8)		233.2 (158.3–559.2)	
TBI/CT− (Ref: TBI/CT+)	146.0 (73.3–286.0)	<0.001	176.1 (93.0–320.3)	<0.001	224.0 (148.1–373.9)	0.2072
All TBI	206.1 (93.9–476.5)		221.2 (120.2–512.7)		230.7 (151.8–491.3)	
OC (Ref:All TBI)	116.9 (78.6–182.6)	<0.001	132.7 (77.2–194.7)	<0.001	167.3 (96.8–285.6)	0.0957
HC (Ref:All TBI)	47.0 (37.0–68.0)	<0.001	74.0 (52.3–96.0)	<0.001	109.0 (75.3–156.8)	<0.001
S100B, ug/L						
TBI/CT+	0.202 (0.105–0.395)		0.178 (0.1–0.414)		0.149 (0.079–0.34)	
TBI/CT− (Ref:TBI/CT+)	0.106 (0.065–0.188)	<0.001	0.107 (0.064–0.176)	<0.001	0.109 (0.078–0.165)	0.024
All TBI	0.131 (0.075–0.258)		0.135 (0.08–0.269)		0.128 (0.078–0.276)	
OC (Ref:All TBI)	0.088 (0.058–0.14)	<0.001	0.084 (0.058–0.125)	<0.001	0.096 (0.076–0.145)	0.281
HC (Ref:All TBI)	0.041 (0.032–0.056)	<0.001	0.051 (0.035–0.066)	<0.001	0.043 (0.035–0.074)	<0.001
NSE, ng/mL						
TBI/CT+	30.54 (19.88–49.53)		24.04 (15.25–46.19)		18.44 (13.45–31.91)	
TBI/CT− (Ref:TBI/CT+)	18.36 (13.74–29.34)	<0.001	17.64 (13.04–26.70)	<0.001	18.64 (14.66–27.74)	0.801
All TBI	22.83 (15.04–39.58)		19.88 (13.83–36.2)		18.46 (13.83–31.48)	
OC (Ref:All TBI)	15.74 (12.16–23.47)	<0.001	15.6 (11.55–20.12)	<0.001	14.99 (12.48–18.88)	0.063
HC (Ref:All TBI)	12.13 (10.27–14.28)	<0.001	12.585 (11.12–15.45)	<0.001	13.715 (11.34–21.74)	0.049

*P*-values are from the Wilcoxon Rank Sum test comparing the biomarkers between diagnostic groups.

Ref = reference group.

In linear regression models, significant interactions between diagnosis group and age category were identified for all day-1 blood-based biomarkers except S100b such that the group difference in these biomarkers decreased with increasing age for CT + versus CT− patients with TBI (interaction term *P*-value = 0.027 for GFAP; *P* < 0.001 for UCH-L1; *P* = 0.047 for NSE) and/or for participants with TBI versus HCs [interaction term *P*-value *P* = 0.090 for GFAP (trend); *P* = 0.038 for UCH-L1; *P* = 0.040 for NSE], but not for participants with TBI versus OCs (all *P*-values >0.3).

**Figure 3:**
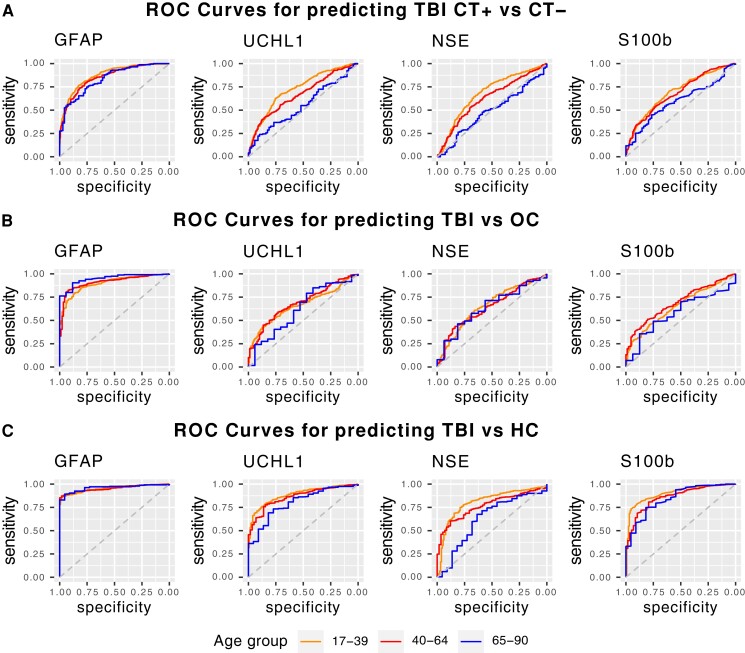
**ROC curves for Day 1 blood-based biomarkers for discrimination of CT+/CT−, TBI/OC, and TBI/HC Stratified by Age.** Each plot shows age-stratified ROC curves of each Day 1 blood-based biomarker for discriminating TBI patients with a positive versus negative head CT (row A), for discriminating TBI patients from orthopedic controls (OC; row B), and for discriminating TBI patients from HCs (row C). ROC curves are colour coded as follows: age 17–39 years is shown in black; age 40–64 years, in red; age 65–90 years, in blue. Specific AUC values with 95% CIs are reported in Table 3.

AUCs of day 1 blood-based biomarkers for discriminating diagnostic groups are reported in Table 3; Fig. 3 shows the ROC curves stratified by age category. Only GFAP had good to excellent AUCs across all age categories for distinguishing all 3 diagnostic groups, with AUCs ranging from 0.84–0.96 and the lower limit of the 95% CIs all >0.78. For all other biomarkers (UCH-L1, NSE, S100B), AUC was statistically significantly lower among older versus young or middle-aged individuals in several diagnostic categories. Performance of UCH-L1 was good for discriminating TBI versus HC among all age categories, fair for discriminating CT + versus CT− among young individuals only but was otherwise poor. Performance of NSE was good for distinguishing TBI versus HC among young individuals, fair for discriminating TBI versus HC among middle-aged individuals but was otherwise poor. Performance of S100B was good to excellent for distinguishing TBI versus HCs across all age-categories but was otherwise poor. Performance of UCHL1 and S100b for discriminating CT-positivity or TBI versus OC was slightly better when the AUC analysis was restricted to samples drawn 0–6 or 7–12 hours post-injury, but performance remained less than good except in the youngest age group which showed good discrimination only of CT + versus CT− at 0–6 h (Supplemental Table 5).

**Table 3:** Discriminative value of day 1 blood-based biomarkers stratified by age

Values are AUC (95% CI)	17–39 years	40–64 years	65–90 years
**GFAP**			
TBI:CT + versus TBI:CT−	**0.874** (**0.854–0.895)**	**0.854** (**0.828–0.88)**	**0.835** (**0.782–0.889)**
TBI versus OC	**0.899** (0.877–0.921)*	**0.911** (**0.884–0.937)**	**0.948** (**0.911–0.985)**
TBI versus HC	**0.954** (**0.943–0.965)**	**0.953** (**0.938–0.967)**	**0.96** (**0.936–0.985)**
**UCH-L1**			
TBI:CT + versus TBI:CT−	0.728 (0.699–0.758)*	0.67 (0.632–0.708)*	0.552 (0.474–0.63)
TBI versus OC	0.663 (0.623–0.703)	0.687 (0.635–0.739)	0.621 (0.474–0.768)
TBI versus HC	**0.886** (**0.862–0.91)**	**0.863** (**0.826–0.901)**	**0.802** (**0.715–0.888)**
**S100B**			
TBI:CT + versus TBI:CT−	0.695 (0.663–0.727)*	0.683 (0.645–0.721)*	0.595 (0.519–0.671)
TBI versus OC	0.652 (0.607–0.697)	0.692 (0.641–0.743)	0.581 (0.453–0.709)
TBI versus HC	**0.902** (**0.877–0.927)**	**0.861** (**0.818–0.905)**	**0.844** (**0.76–0.927)**
**NSE**			
TBI:CT + versus TBI:CT−	0.679 (0.647–0.712)*	0.626 (0.586–0.667)*	0.489 (0.409–0.57)
TBI versus OC	0.654 (0.605–0.702)	0.642 (0.584–0.7)	0.639 (0.507–0.772)
TBI versus HC	**0.806** (0.768–0.844)*	0.769 (0.722–0.816)*	0.627 (0.496–0.758)

**Bolded result** indicates AUC at least 0.8 (at least ‘good’ discrimination).

* *P* < 0.05 versus 65–90 years.

### Day 1 GFAP, UCHL-1 and S100B cut-off analysis

Cut-off analysis of day 1 GFAP 30 pg/ml, UCHL-1 360 pg/ml and S100B 0.1 ug/L to identify CT-positivity are shown in Table 4, stratified by age and sampling time-interval. For GFAP, sensitivity is >95% and negative predictive value (NPV) is >91% across all age categories and time-intervals. Among older adults in particular, GFAP >30 pg/ml had 100% sensitivity and NPV for CT-positivity. For UCHL-1, sensitivity is <90% in all sub-categories and NPV only reaches 90%+ among young individuals (but not middle-aged or older adults) at 0–6 h post-injury (NPV 0.96). For S100B, sensitivity and NPV are only >90% among young and middle-aged individuals (but not older adults) at 0–6 h post-injury. Specificity, however, was very low for GFAP (<0.40 at all timepoints and across all age categories) and S100B (<0.58 at all timepoints and across all age categories) but much higher for UCHL-1, especially at 13–12 h post-injury (range 0.71–0.85 across age categories).

**Table 4:** Cut-off analysis of day 1 GFAP, UCHL-1 and S100b to identify CT-positivity stratified by age and sampling time-interval

Sampling-Interval	Sens (95% CI)	Spec (95% CI)	NPV (95% CI)	PPV (95% CI)
**GFAP cut-off 30 pg/ml**				
**Age 17–39** **years**				
0–6 hours	**0.957** (**0.870–1)**	0.396 (0.319–0.479)	**0.983** (**0.944–1)**	0.202 (0.177–0.228)
7–12 hours	**0.989** (**0.968–1)**	0.199 (0.135–0.263)	**0.97** (**0.893–1)**	0.429 (0.411–0.449)
13–24 hours	**0.985** (**0.971–0.997)**	0.278 (0.232–0.324)	**0.957** (**0.913–0.99)**	0.544 (0.529–0.56)
0–24 hours	**0.985** (**0.974–0.996)**	0.285 (0.251–0.316)	**0.967** (**0.940–0.989)**	0.478 (0.467–0.49)
**Age 40–64** **years**				
0–6 hours	**0.971** (**0.886–1)**	0.344 (0.258–0.441)	**0.972** (**0.892–1)**	0.359 (0.327–0.398)
7–12 hours	**0.986** (**0.957–1)**	0.161 (0.081–0.258)	**0.917** (**0.714–1)**	0.567 (0.542–0.596)
13–24 hours	**0.986** (**0.972–0.997)**	0.193 (0.142–0.25)	**0.914** (**0.824–0.98)**	0.623 (0.608–0.64)
0–24 hours	**0.985** (**0.972–0.995)**	0.226 (0.183–0.267)	**0.935** (**0.88–0.978)**	0.576 (0.562–0.59)
**Age 65–90** **years**				
0–6 hours	**1** (**1–1)**	0.053 (0–0.159)	**1** (**1–1)**	0.308 (0.296–0.334)
7–12 hours	**1** (**1–1)**	0.231 (0–0.462)	**1** (**1–1)**	0.737 (0.683–0.8)
13–24 hours	**1** (**1–1)**	0.081 (0–0.189)	**1** (**1–1)**	0.799 (0.785–0.818)
0–24 hours	**1** (**1–1)**	0.101 (0.029–0.174)	**1** (**1–1)**	0.734 (0.718–0.75)
**UCH-L1 cut-off 360 pg/ml**				
**Age 17–39** **years**				
0–6 hours	0.826 (0.652–0.957)	0.736 (0.660–0.806)	**0.964** (**0.932–0.991)**	0.333 (0.264–0.417)
7–12 hours	0.695 (0.600–0.789)	0.718 (0.647–0.788)	0.796 (0.743–0.847)	0.600 (0.531–0.673)
13–24 hours	0.423 (0.370–0.475)	0.867 (0.834–0.903)	0.631 (0.611–0.657)	0.737 (0.680–0.797)
0–24 hours	0.499 (0.451–0.542)	0.806 (0.776–0.832)	0.707 (0.688–0.726)	0.631 (0.592–0.669)
**Age 40–64** **years**				
0–6 hours	0.629 (0.457–0.771)	0.634 (0.538–0.731)	0.819 (0.747–0.887)	0.392 (0.298–0.484)
7–12 hours	0.609 (0.493–0.725)	0.823 (0.710–0.903)	0.654 (0.587–0.727)	0.794 (0.698–0.88)
13–24 hours	0.415 (0.362–0.470)	0.849 (0.802–0.892)	0.517 (0.49–0.544)	0.787 (0.732–0.847)
0–24 hours	0.468 (0.417–0.519)	0.790 (0.749–0.831)	0.582 (0.557–0.611)	0.703 (0.660–0.750)
**Age 65–90** **years**				
0–6 hours	0.625 (0.25–0.875)	0.579 (0.368–0.789)	0.786 (0.625–0.938)	0.385 (0.200–0.583)
7–12 hours	0.429 (0.25–0.607)	0.692 (0.462–0.923)	0.357 (0.24–0.481)	0.750 (0.562–0.929)
13–24 hours	0.341 (0.267–0.422)	0.784 (0.649–0.919)	0.246 (0.207–0.284)	0.852 (0.766–0.932)
0–24 hours	0.368 (0.298–0.439)	0.710 (0.609–0.812)	0.313 (0.269–0.352)	0.760 (0.677–0.833)
**S100B cut-off 0.1** **ug/l**				
**Age 17–39** **years**				
0–6 hours	**0.952** (**0.857–1)**	0.400 (0.319–0.481)	**0.983** (**0.940–1)**	0.198 (0.173–0.226)
7–12 hours	0.867 (0.789–0.933)	0.331 (0.254–0.408)	0.800 (0.703–0.889)	0.451 (0.419–0.488)
13–24 hours	0.736 (0.688–0.781)	0.557 (0.512–0.608)	0.704 (0.665–0.745)	0.597 (0.568–0.630)
0–24 hours	0.773 (0.734–0.811)	0.477 (0.440–0.515)	0.755 (0.721–0.789)	0.501 (0.48–0.524)
**Age 40–64** **years**				
0–6 hours	**0.906** (**0.781–1)**	0.299 (0.207–0.391)	**0.900** (**0.778–1)**	0.323 (0.282–0.360)
7–12 hours	0.821 (0.716–0.896)	0.356 (0.254–0.475)	0.634 (0.500–0.784)	0.590 (0.543–0.648)
13–24 hours	0.714 (0.663–0.765)	0.576 (0.512–0.649)	0.600 (0.548–0.652)	0.695 (0.658–0.733)
0–24 hours	0.749 (0.707–0.792)	0.470 (0.419–0.521)	0.637 (0.594–0.685)	0.602 (0.577–0.629)
**Age 65–90** **years**				
0–6 hours	0.875 (0.625–1)	0.368 (0.158–0.579)	0.875 (0.666–1)	0.368 (0.273–0.500)
7–12 hours	0.760 (0.560–0.920)	0.417 (0.167–0.667)	0.455 (0.214–0.715)	0.731 (0.630–0.840)
13–24 hours	0.628 (0.543–0.713)	0.459 (0.297–0.622)	0.262 (0.182–0.350)	0.802 (0.753–0.857)
0–24 hours	0.660 (0.586–0.735)	0.426 (0.324–0.544)	0.347 (0.271–0.427)	0.734 (0.690–0.779)

**Bolded result** indicates sensitivity (sens), specificity (spec), NPV, or positive predictive value (PPV) at least 0.90.

### Longitudinal biomarker cohort results

**Figure 4:**
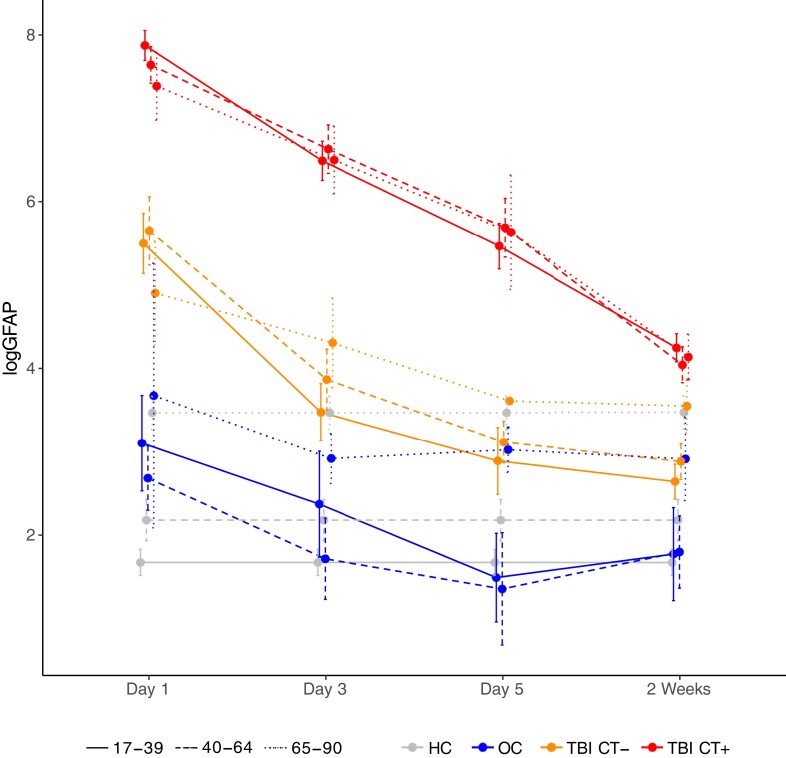
**Longitudinal (Day 1 through week 2) blood-based GFAP levels stratified by diagnostic group and age.** Mean with 95% CIs is shown for log-transformed GFAP levels stratified by age category and diagnostic group, with the same colour coding as in Fig. 3. HC biomarker levels were only drawn at a single time-point so the same values are plotted here repeatedly for comparison to the other diagnostic groups over time. Specific blood-biomarker levels and results of Wilcoxon rank sum tests comparing levels across diagnostic groups, stratified by age and days post-injury, are reported in Supplemental Table 7.

Baseline demographic and clinical characteristics of the longitudinal TBI cohort stratified by age category are shown in Supplemental Table 6. Clinical characteristics are similar to the overall cohort except that injury severity is greater because all participants in the longitudinal cohort were admitted to the hospital. Median and IQR for each blood-based biomarker level on Day 1, Day 3, Day 5 and Week 2 by diagnostic group and age category are shown in Supplemental Table 7. Log-transformed means of longitudinal GFAP are plotted in Fig. 4 (and log-transformed means of longitudinal UCHL1, NSE and S100b are plotted in Supplemental Figures 2–4, respectively). While GFAP levels gradually decline among TBI participants over the 2 weeks post-injury, GFAP levels remain significantly higher among TBI participants with CT + versus CT−, among TBI versus OCs, and among TBI versus HCs across all age groups (except on day 5 there are too few CT− older adults to comment on this group difference) . Although GFAP levels among HCs and OCs are impacted by age, levels among those with TBI, particularly TBI/CT + do not appear to be impacted by age as all ages experience a remarkably similar magnitude of acute elevation with remarkably similar rates of decline over 2 weeks Levels of UCH-L1, S100B, and NSE decline more rapidly among TBI participants after day 1 in all age groups.

AUCs of longitudinal GFAP for discriminating diagnostic groups are reported in Table 5 stratified by age category. Among young adults, AUCs were good to excellent out to 2 weeks for discriminating all diagnostic groups. Among middle-aged adults, AUCs were good to excellent out to day 5 for discriminating CT + versus CT− and out to 2 weeks for discriminating TBI from OC or HC. For older adults, AUCs were good to excellent out to 3 days for discriminating CT + versus CT−, out to 5 days for discriminating TBI versus HC and out to 2 weeks for discriminating TBI versus OC. AUCs of longitudinal UCH-L1, S100B, and NSE for discriminating diagnostic groups are reported in Supplemental Table 8 stratified by age category and are overall only fair to poor (AUCs <0.8) beyond Day 1 with only isolated exceptions of uncertain clinical relevance.

**Table 5:** Discriminative value of Day 1, Day 3, Day 5, and Week 2 GFAP stratified by age

Values are AUC (95% CI)	17–39 years	40–64 years	65–90 years
TBI/CT + versus TBI/CT−			
Day 1	**0.882** (**0.839–0.925)**	**0.843** (**0.785–0.901)**	**0.911** (**0.838–0.983)**
Day 3	**0.921** (**0.883–0.959)**	**0.896** (**0.851–0.941)**	**0.895** (**0.815–0.975)**
Day 5	**0.912** (**0.859–0.964)**	**0.913** (**0.863–0.963)**	**NA (only *N* = 1 TBI/CT−)**
Week 2	**0.869** (**0.824–0.913)**	0.781 (0.716–0.847)	0.714 (0.573–0.855)
TBI versus OC			
Day 1	**0.958** (**0.932–0.985)**	**0.984** (**0.969–1)**	**0.905** (**0.744–1)**
Day 3	**0.911** (**0.860–0.962)**	**0.979** (**0.957–1)**	**0.975** (**0.935–1)**
Day 5	**0.968** (**0.941–0.995)**	**0.990** (**0.969–1)**	**0.919** (**0.826–1)**
Week 2	**0.897** (**0.843–0.951)**	**0.936** (**0.892–0.979)**	**0.875** (**0.774–0.976)**
TBI versus HC			
Day 1	**0.989** (**0.979–0.998)**	**0.991** (**0.982–1)**	**0.965** (**0.927–1)**
Day 3	**0.950** (**0.929–0.972)**	**0.955** (**0.932–0.979)**	**0.942** (**0.892–0.993)**
Day 5	**0.937** (**0.908–0.966)**	**0.929** (**0.895–0.963)**	**0.867** (**0.759–0.976)**
Week 2	**0.901** (**0.872–0.930)**	**0.854** (**0.805–0.903)**	0.688 (0.575–0.802)

**Bolded result** indicates AUC at least 0.8 (at least ‘good’ discrimination).

## Discussion

In this large multi-centre study of day-1 through week-2 TBI diagnostic blood-based biomarkers among patients presenting to a trauma centre within 24 hours of all-severity TBI, plasma GFAP measurement had good to excellent discrimination of important diagnostic groups up to at least 3 days post-injury among older adults, up to 5 days post-injury among middle-aged adults and up to 2 weeks post-injury among young adults. S100B showed good discrimination of participants with TBI from HCs up to 24 hours post-injury across the adult age spectrum. UCHL1 showed good discrimination of participants with TBI from HCs up to 12 hours post-injury among older adults and up to 24 hours post-injury among young and middle-aged adults. For the more challenging discrimination of CT + versus CT− participants with TBI, UCHL-1 and S100B performed well only among adults under age 40 years and only up to 6 hours post-injury. For discrimination of TBI from OC, UCHL-1 and S100B performed fair to poorly across all ages and time-intervals. NSE mostly performed fair to poorly except for discriminating TBI from HC among the youngest adults.

We also identified a significantly smaller difference in blood-based biomarker levels between diagnostic groups (i.e. between CT + versus CT− and between TBI versus HC) among older adults compared to younger adults. This important finding effectively translates into a narrower range within which a TBI diagnosis may be discriminated among older adults as well as a more rapid attenuation of differences over time. This finding is in line with several prior studies of brain injury biomarkers,^23,35,36^ that reported that older adults tend to have higher baseline levels of brain injury biomarkers in the absence of injury and lower acute elevations after brain injury compared with younger adults. This age-related baseline elevation impacted GFAP most prominently, followed by UCHL1, in our study.

Cut-off analysis based on established diagnostic thresholds for the iSTAT GFAP and iSTAT UCHL1 assays^33^ and the Roche Elecsys S100b assay for discriminating CT-positivity^34^ confirmed excellent (at least 0.9) sensitivity and NPV for GFAP across all age groups throughout day 1, excellent sensitivity and NPV for S100b among young and middle-aged (but not older) adults up to 6 hours post-injury, and excellent NPV for UCHL1 only among young adults (but not middle-aged or older) up to 6 hours post-injury. This age-related drop in sensitivity and NPV was partly due to the rising prevalence of CT positivity with increasing age but also due to the declining accuracy of UCHL1 and S100B with increasing age seen in the day 1 ROC analysis and demonstrates the importance of considering age-related differences when establishing and interpreting diagnostic thresholds. Overall, our findings confirm and extend the scope of GFAP as a promising blood-based TBI diagnostic for adults of all ages.

Our prior smaller study,^23^ as well as four other prior studies, investigated the effect of older age on performance of blood-based TBI diagnostic biomarkers.^19–22^ In our prior study of 169 patients presenting within 24 hours of mild TBI (GCS 13–15), we found that a GFAP ELISA assay was significantly less accurate for identifying intracranial trauma on head CT among older adults (AUC 0.73) compared with young or middle-aged adults (AUC 0.92–0.93).^23^ Similarly, three large studies of patients presenting within 3 hours of mild TBI (GCS 14–15) studied accuracy of an S100b assay to discriminate CT + from CT− patients.^19–21^ These studies reported 95–100% sensitivity across all ages, but very low specificity, that was even lower among older patients, suggesting that S100B will not save many head CTs in the elderly population unless age-specific thresholds are developed.^19^ Similarly, a *post hoc* analysis of 1959 patients presenting within 12 hours of mild to moderate head injury (GCS 9–15) who participated in the ALERT-TBI study reported 100% sensitivity and NPV for a combined GFAP ELISA/UCH-L1 ELISA assay for predicting absence of intracranial trauma on head CT among both older and younger adults, but very poor specificity and PPV among older adults.^22^ Of note, in the ALERT-TBI trial, less than 10% of patients had a positive head CT and patients presented within 12 hours of injury. Together, this prior body of evidence suggests that TBI blood-based biomarkers may be highly sensitive for detecting CT positivity across all ages, but raises concerns about specificity. Our current study adds to the existing literature by identifying improved performance of a higher sensitivity GFAP assay (LoD <15 pg/mL) for detecting CT positivity in older adults compared with the lower sensitivity GFAP assay (LoD 100 pg/mL) used in our prior study^23^ and by the ALERT-TBI investigators. Our current study additionally confirms good accuracy of S100b and UCHL1 within 6 hours of injury for detecting CT positivity among young adults only (but not middle-aged or older adults) and within 24 hours of injury for discriminating TBI from HC among all ages. The superiority of GFAP over S100b, UCHL1, and NSE for discriminating CT positive from CT negative patients presenting within 24 hours of TBI confirms the prior findings from the Collaborative European Neuro-Trauma Effectiveness Research (CENTER-TBI) Core study.^37^ This prior CENTER-TBI study reported that plasma GFAP was more accurate than plasma S100b, UCHL1, NSE, neurofilament light, or tau for discriminating CT + from CT− individuals presenting within 24 hours of TBI; however, their study did not stratify by age.^37^

The above-mentioned prior studies of blood-based TBI diagnostic biomarkers did not investigate timepoints beyond 3–24 hours or discrimination of TBI patients from controls. Our current study therefore substantially extends this prior work by investigating additional diagnostic groups (e.g. HCs and OCs) and longitudinal performance of these four emerging biomarkers out to 2 weeks post-injury. However, while our study did investigate biomarker levels beyond day 1 post-injury via serial sampling in a subset of hospitalized participants, our cohort included only patients who initially presented to a level 1 trauma centre within 24 hours of injury. Since many elderly individuals present for medical care days after a fall, further research is warranted to determine whether this GFAP assay may have value for supporting the outpatient diagnosis of TBI among patients presenting several days post-injury to an urgent care or clinic setting in whom the diagnosis of TBI is in question. In these future studies, age effects should be specifically investigated given our demonstration of more rapid attenuation of differences in GFAP levels across diagnostic groups among older versus younger adults.

We also examined NSE as a blood-based TBI diagnostic biomarker. As shown in Supplemental Fig. 3, NSE demonstrates an initial decline between day 1 and day 3 followed by a delayed elevation in some TBI patients across all age groups between day 5 and week 2 post-injury. While numbers are small and this finding of delayed NSE elevation should therefore be considered exploratory, this pattern was not observed in any of the other biomarkers which all demonstrated fairly linear decline plus/minus plateau post-injury At least 1 prior study reported chronic elevations in blood NSE among special operations forces combat soldiers with a history of TBI compared to soldiers without a history of TBI, supporting a potential role for NSE as a marker of prior/chronic TBI.^38^ Future studies investigating associations of longitudinal NSE with different TBI pathoanatomical subtypes, neurobehavioral outcomes, and functional recovery are warranted.

Currently, S100B, GFAP and UCH-L1 are at varying stages of implementation in clinical practice. Plasma S100B is included in the Scandinavian TBI guidelines to aid in the decision to defer head CT in patients presenting within 6 hours of injury with mild TBI, except for patients age > 65 years who are taking anti-platelet agents, in whom head CT was considered indicated regardless of symptoms or S100B level. Plasma GFAP and UCHL-1 are approved by the U.S. FDA to aid in the evaluation of patients age ≥18 years presenting within 12 hours of suspected mild TBI defined as GCS 13–15 based largely on the ALERT-TBI trial.^14^ FDA subsequently approved the i-STAT® System handheld device, used in this current study, for measurement of blood-based GFAP and UCH-L1.^17,26^ Our findings generate the hypothesis that GFAP may be valuable for TBI diagnosis amongst older adults presenting up to 3 days post-injury, among middle aged adults presenting up to 5 days post-injury, and among young adults presenting up to 2 weeks post-injury, but further research is needed in patients presenting for care in a delayed fashion as discussed above.

This study has many strengths. This is among the largest, multi-centre, prospective cohort study to date that has correlated age and time since injury with these four leading blood-based TBI diagnostic biomarkers. This study included participants across a wide age range: from age 17 to 90 years. This study included not only elderly individuals with TBI but also elderly orthopedic controls and elderly HCs. Limitations include the small sample sizes in the age-stratified cohorts, particularly in the age-stratified longitudinal cohorts, and thus our findings warrant replication in even larger samples. Day 3, 5 and 14 samples were obtained only in a subset of participants with relatively more severe injuries who presented within 24 hours but required hospitalization; thus, our longitudinal results may not accurately reflect patients who present for medical attention days after injury. Due to small samples after stratification, we were unable to investigate interactions with specific neuroimaging features. For example, further research is needed to determine performance of blood-based TBI biomarkers in the detection of isolated subdural haemorrhage in older patients in which brain parenchymal injury may be minimal. Because the OCs in this study did not undergo head CT, it is possible that they sustained an occult TBI that was missed clinically, for all of the reasons outlined in the introduction. However, elevations in GFAP, UCHL-1 and S100B have also been described to occur after orthopedic trauma/fracture without TBI.^39,40^ Additionally, because GFAP levels among OCs were observed to ultimately fall below those of HCs among middle-aged and older adults, this suggests that there were baseline neurological differences between these two groups of controls that may have introduced bias into this analysis. This hypothesis is further supported by the slightly higher prevalence of stroke/TIA, prior TBI and prior psychiatric illness observed in the older ‘friend’ HCs versus the older OCs reported in Supplemental Tables 1 and 2, despite being slightly younger in age. Prevalence of these conditions in the ‘community’ HCs is unknown; however, the ‘community’ HCs were somewhat older than the OCs (mean age 75.6 versus 71.3) suggesting that they likely do have higher prevalence of these pre-existing neurological disorders. These observations highlight some of the challenges of using non-specific brain biomarkers to discriminate a diagnosis of TBI in older adults who may have multiple pre-existing neurological disorders.

Lastly, because participants with dementia were excluded from this study, it is unknown whether our findings will generalize to this important geriatric TBI sub-population who may comprise 11–24% of older adults presenting to trauma centres with acute TBI.^19,41^ One of the prior studies of S100b elevations in geriatric TBI mentioned above identified significantly higher acute elevations in S100b among older adults presenting with mTBI who had dementia versus those without dementia.^19^ The relationship between age, dementia, TBI, and blood-based GFAP may prove particularly complex. One elegant prior study with detailed assessment of both cognitive status and cerebral amyloid burden on amyloid PET scan identified a positive correlation between blood-based GFAP levels and amyloid burden among cognitively normal older adults, suggesting that in early/pre-clinical stages of Alzheimer’s disease, blood-based GFAP levels may be elevated.^42^ In the same study, however, patients with severe Alzheimer’s dementia showed a negative association between blood-based GFAP and amyloid burden such that those with higher amyloid burden had lower GFAP levels, suggesting that as neurodegeneration and cell death progress, GFAP levels ultimately drop. Blood biomarker studies such as the ongoing Transforming Research And Clinical Knowledge in Geriatric TBI study—a TRACK-TBI network study that is currently enrolling adults aged 65 years and older, inclusive of those with pre-existing cognitive impairment and dementia—will be important for determining generalizability to all older adults.

This study highlights the importance of considering the effects of age on the diagnostic performance of blood-based TBI biomarkers. It also supports the importance of continuing to develop and test ultra-high-sensitivity blood-based TBI biomarkers which may ultimately be able to overcome age-related decrements in performance, as demonstrated here for GFAP. Replication of our findings in additional longitudinal cohorts and to comprehensively define the breadth of appropriate use in children, the pre-hospital setting and in patients of all ages with milder TBI presenting several days post-injury will be important. In conclusion, the addition of a blood-based TBI diagnostic to the evaluation of TBI—including geriatric TBI—has the potential to substantially streamline patient triage, reduce delays in appropriate management, reduce unnecessary CT scans and decrease healthcare costs in this rapidly growing population.

## Supplementary Material

fcac316_Supplementary_Data

## Data Availability

Baseline clinical data for the current analysis are from the frozen TRACK-TBI U01 data-set from 06/22/21 which were then merged with the TBI and OC blood biomarker data-set from 4/07/21 and the HC blood biomarker data-set from 5/30/18. De-identified data are available after submission of a data use agreement to the TRACK-TBI Executive Committee or by request from the Federal Interagency TBI Research Informatics System.^43^

## Appendix I

### The TRACK-TBI investigators

Venkata R. Feeser, Adam R. Ferguson, Etienne Gaudette, Shankar Gopinath, C. Dirk Keene, Christopher Madden, Alastair Martin, Michael McCrea, Randall Merchant, Pratik Mukherjee, Laura B. Ngwenya, Claudia Robertson, Nancy Temkin, Mary Vassar, John K. Yue and Ross Zafonte.

## Abbreviations

AUC =: area under the curve
CENTER-TBI =: Collaborative European Neuro-Trauma Effectiveness Research
CI =: confidence interval
CV =: coefficient of variation
FDA =: U.S. Food and Drug Administration
GCS =: Glasgow Coma Scale
GFAP =: glial fibrillary acidic protein
HC =: healthy control
LoD =: limit of detection
LoQ =: limit of quantification
NPV =: negative predictive value
NSE =: neuron-specific enolase
OC =: orthopedic trauma control
ROC =: receiver operating characteristic
RR =: reportable range
S100B =: S100 calcium binding protein B
TBI =: traumatic brain injury
TRACK-GERI =: Transforming Research And Clinical Knowledge in Geriatric TBI
TRACK-TBI =: Transforming Research and Clinical Knowledge in TBI
U.S. =: United States
UCH-L1 =: ubiquitin carboxy-terminal hydrolase L1
