# Supplementary material for: Effects of age and time since injury on traumatic brain injury blood biomarkers: a TRACK-TBI study
Source: Brain Commun. 2022 Dec 1;5(1):fcac316. doi: 10.1093/braincomms/fcac316 (PMC9832515; doi:10.1093/braincomms/fcac316)
Supplement: fcac316_Supplementary_Data [file fcac316_supplementary_data.pdf]

Supplementary Material

Supplemental Table 1. Characteristics of Orthopedic Controls by Age Group

**Characteristic**<br>N(%) or mean(SD)	**17-39y**<br>**N=136**	**40-64y**<br>**N=89**	**65-90y**<br>**N=17**	**p-value**
**Age (years)**	28.4 ±5.8	51.5 ±7.4	71.3 ±5.1	<0.001
**Female**	42 (30.88%)	34 (38.2 %)	9 (52.94%)	0.154
**Race**				0.316
White	99 (75.57%)	74 (84.09%)	14 (82.35%)	
Black	23 (17.56%)	11 (12.5%)	1 (5.88%)	
Other	9 (6.87%)	3 (3.41%)	2 (11.76%)	
**Hispanic**	38 (28.57%)	17 (19.32%)	3 (18.75%)	0.274
**Education (years)**	13.8±2.5	13.6 ±3.0	15.2 ±3.3	0.061
**Past medical history**				
HTN	3 (2.21%)	22 (24.72%)	10 (58.82%)	<0.001
Hyperlipidemia	1 (0.74%)	2 (2.25%)	4 (23.53%)	<0.001
Ischemic heart disease	0 (0%)	0 (0%)	0 (0%)	-
Stroke or TIA	0 (0%)	2 (2.25%)	0 (0%)	0.261
Diabetes	3 (2.21%)	10 (11.24%)	3 (17.65%)	0.003
Renal disease	4 (2.94%)	4 (4.49%)	1 (5.88%)	0.555
Pulmonary disease	23 (16.91%)	9 (10.11%)	4 (23.53%)	0.192
Prior TBI	33 (25.38%)	21 (24.42%)	1 (5.88%)	0.192
**Psychiatric history**	34 (25.0%)	26 (29.21%)	3 (17.65%)	0.617
**Disposition**				0.015
ED discharge	59 (43.38%)	27 (30.34%)	2 (11.76%)	
Hospital ward admit	68 (50%	55 (61.8%)	11 (64.71%)	
ICU admit	9 (6.62%)	7 (7.87%)	4 (23.53%)	
**Blood draw time post-injury, hours**	12.6 ±7.2	14.0 ±7.7	15.2 ±6.7	0.155

Supplemental Table 2. Demographics of "Friend" Healthy Controls by Age Group

Characteristic<br>N(%) or mean (standard deviation)	17-39y<br>N=67	40-64y<br>N=31	65-90y<br>N=6	p-value
**Age (years)**	27.2 ±5.6	51.0 ±6.1	69.2±3.9	<0.001
**Female**	33 (49.3%)	25 (80.7%)	4 (66.7%)	0.009
**Race**				
White	53 (80.3%)	26 (83.9%)	5 (83.3%)	0.896
Black	8 (12.1%)	4 (12.9%)	1 (16.7%)	
Other	5 (7.6%)	1 (3.2%)	0 (0%)	
**Hispanic**	14 (21.2%)	4 (12.9%)	0 (0%)	0.458
**Education (years)**	14.1±2.0	13.7±2.7	15.3±2.0	0.133
**Past medical history**				
Hypertension	2 (3.0%)	4 (12.9%)	3 (50%)	0.005
Hyperlipidemia	0 (0%)	0 (0)%)	0 (0%)	-
Ischemic heart disease	0 (0%)	0 (0)%)	0 (0%)	-
Stroke or transient ischemic attack	0 (0%)	0 (0%)	1 (16.7%)	0.061
Diabetes	1 (1.5%)	1 (3.2%)	1 (16.7%)	0.121
Renal disease	2 (3.0%)	1 (3.2%)	0 (0%)	>0.999
Pulmonary disease	12 (17.9%)	7 (22.6%)	2 (33.3%)	0.505
Prior traumatic brain injury	22 (32.8%)	8 (25.8%)	2 (33.3%)	0.790
**Psychiatric history**	13 (19.4%)	12 (38.7%)	3 (50%)	0.039

Supplemental Table 3. Demographics of "Community" Healthy Controls by Age Group

Characteristic<br><br>N(%) or mean (standard deviation)	**17-39y**<br><br>**N=63**	**40-64y**<br><br>**N=26**	**65-90y**<br><br>**N=16**	**p-value**
**Age (years)**	27.8 ±5.7	52.1 ±7.3	75.6 ±7.0	<0.001
**Female**	24 (38.1%)	9 (34.6%)	8 (50%)	0.577

Supplemental Table 4. Day 1 blood-based biomarkers levels stratified by diagnostic group, age, and sampling time interval

Values are median (interquartile range)	**17-39y**	**P-value**	**40-64y**	**P-value**	**65-90y**	**P-value**
**GFAP, pg/mL**						
**0-6h**						
TBI/CT+	1281.1 (522.2-7976.6)		1026.6 (264.7-2229.0)		912.0 (378.7-3072.9)**	
TBI/CT- (Ref:TBI/CT+)	57.4 (12,4-213.2)	<0.001	82.7 (17.4-244.2)	<0.001	87.9 (47.5-163.0)*	(<0.001)
All TBI	86.3 (14.6-377.9)		171.0 (26.5-504.8)		110.0 (74.0-705.9)	
OC (Ref:All TBI)	6.9 (3.6-12.2)	<0.001	14.7 (7.6-23.0)	<0.001	98.2 (56.4-140.0)**	(0.443)
HC (Ref:All TBI)	5.0 (2.0-10.0)	<0.001	10.0 (5.0-15.0)	<0.001	28.5 (25.3-38.8)	<0.001
**7-12h**						
TBI/CT+	1680.7 (735.6-3842.2)		1805.5 (671.0-3540.0)		543.1 (247.8-3040.3)	
TBI/CT- (Ref:TBI/CT+)	184.6 (54.7-573.0)	<0.001	317.9 (64.1-698.0)	<0.001	115.2 (38.5-379.2)*	0.004
All TBI	473.3 (100.6-1563.0)		705.4 (158.7-2132.5)		351.8 (211.5-1718.1)	
OC (Ref:All TBI)	11.0 (5.3-32.8)	<0.001	13.2 (9.0-45.0)*	<0.001	38.1 (22.0-47.0)**	(<0.001)
HC (Ref:All TBI)	5.0 (2.0-10.0)	<0.001	10.0 (5.0-15.0)	<0.001	28.5 (25.3-38.8)	<0.001
**13-24h**						
TBI/CT+	1609.8 (596.0-4161.3)		1520.6 (546.3-3240.3)		1197.0 (430.9-3351.3)	
TBI/CT- (Ref:TBI/CT+)	142.6 (22.6-433.9)	<0.001	190.4 (49.7-447.7)	<0.001	242.6 (54.2-464.4)	<0.001
All TBI	460.6 (95.1-1720.5)		630.6 (166.5-2090.4)		833.3 (294.0-2675.6)	
OC (Ref:All TBI)	8.0 (3.3-21.4)	<0.001	14.4 (7.5-26.5)	<0.001	25.5 (16.1-41.0)*	<0.001
HC (Ref:All TBI)	5.0 (2.0-10.0)	<0.001	10.0 (5.0-15.0)	<0.001	28.5 (25.3-38.8)	<0.001
**UCH-L1, pg/mL**						
**0-6h**						
TBI/CT+	934.2 (541.3-1805.4)		657.6 (279.9-1483.3)		688.1 (215.1-1350.2)**	
TBI/CT- (Ref:TBI/CT+)	216.5 (114.4-382.1)	<0.001	237.6 (159.6-461.0)	<0.001	256.8 (184.9-567.2)*	(0.163)
All TBI	242.0 (123.7-538.5)		308.3 (163.1-726.6)		348.8 (185.1-602.2)	
OC (Ref:All TBI)	114.0 (78.8-170.3)	<0.001	139.8 (97.5-209.0)	<0.001	1638.3 (956.0-2320.6)**	(0.315)
HC (Ref:All TBI)	47.0 (37.0-68.0)	<0.001	74.0 (52.3-96.0)	<0.001	109.0 (75.3-156.8)	<0.001
**7-12h**						
TBI/CT+	737.7 (305.1-1192.9)		554.1 (206.0-1423.0)		323.9 (190.1-1134.8)	
TBI/CT- (Ref:TBI/CT+)	190.8 (88.7-415.1)	<0.001	216.7 (114.6-307.3)	<0.001	295.0 (154.8-373.9)*	0.298
All TBI	284.0 (120.4-795.6)		290.7 (141.5-679.1)		300.8 (186.5-614.0)	
OC (Ref:All TBI)	213.9 (138.6-299.4)	0.104	169.6 (77.4-244.8)*	0.016	285.6 (141.6-442.7)**	(0.408)
HC (Ref:All TBI)	68.0 (47.0-356.0)	<0.001	74.0 (52.3-96.0)	<0.001	109.0 (75.3-156.8)	<0.001
**13-24h**						
TBI/CT+	314.7 (139.3-567.0)		277.3 (139.7-686.0)		227.1 (148.8-462.1)	
TBI/CT- (Ref:TBI/CT+)	107.7 (58.0-2132.3)	<0.001	146.4 (82.7-246.8)	<0.001	183.8 (129.0-304.2)	0.100
All TBI	169.4 (81.0-376.7)		199.0 (107.7-427.0)		221.4 (137.0-432.9)	
OC (Ref:All TBI)	102.7 (68.2-150.9)	<0.001	119.8 (76.5-170.6)	<0.001	115.7 (95.2-183.7)*	0.023
HC (Ref:All TBI)	47.0 (37.0-68.0)	<0.001	74.0 (52.3-96.0)	<0.001	109.0 (75.3-156.8)	<0.001

**S100B, ug/L**						
**0-6h**						
TBI/CT+	0.37 (0.23-0.61)		0.40 (0.23-0.73)		0.25 (0.20-0.46)**	
TBI/CT- (Ref:TBI/CT+)	0.12 (0.08-0.23)	<0.001	0.15 (0.09-0.27)	<0.001	0.19 (0.08-0.33)*	(0.441)
All TBI	0.14 (0.08-0.27)		0.21 (0.11-0.37)		0.23 (0.09-0.33)	
OC (Ref:All TBI)	0.09 (0.06-0.16)	0.008	0.09 (0.06-0.13)	<0.001	0.46 (0.32-0.61)**	(0.519)
HC (Ref:All TBI)	0.04 (0.03-0.06)	<0.001	0.05 (0.04-0.07)	<0.001	0.04 (0.04-0.07)	<0.001
**7-12h**						
TBI/CT+	0.30 (0.16-0.54)		0.23 (0.12-0.56)		0.16 (0.11-0.53)	
TBI/CT- (Ref:TBI/CT+)	0.13 (0.08-0.22)	<0.001	0.14 (0.08-0.18)	<0.001	0.12 (0.08-0.16)*	0.072
All TBI	0.17 (0.10-0.31)		0.16 (0.10-0.29)		0.14 (0.09-0.36)	
OC (Ref:All TBI)	0.12 (0.09-0.19)*	0.054	0.12 (0.08-0.15)*	0.024	0.13 (0.11-0.13)**	(0.683)
HC (Ref:All TBI)	0.04 (0.03-0.06)	<0.001	0.05 (0.04-0.07)	<0.001	0.04 (0.04-0.07)	<0.001
**13-24h**						
TBI/CT+	0.18 (0.10-0.35)		0.16 (0.09-0.38)		0.14 (0.07-0.28)	
TBI/CT- (Ref:TBI/CT+)	0.09 (0.06-0.16)	<0.001	0.09 (0.06-0.14)	<0.001	0.10 (0.07-0.14)	0.022
All TBI	0.12 (0.07-0.24)		0.12 (0.08-0.23)		0.12 (0.07-0.25)	
OC (Ref:All TBI)	0.07 (0.05-0.12)	<0.001	0.08 (0.06-0.11)	<0.001	0.08 (0.07-0.09)**	(0.077)
HC (Ref:All TBI)	0.04 (0.03-0.06)	<0.001	0.05 (0.04-0.07)	<0.001	0.04 (0.04-0.07)	<0.001
**NSE, ng/mL**						
**0-6h**						
TBI/CT+	29.7 (19.7-65.7)		22.0 (16.3-42.0)		29.4 (16.4-42.2)**	
TBI/CT- (Ref:TBI/CT+)	16.5 (13.9-23.8)	<0.001	17.0 (12.7-26.8)	0.026	21.7 (15.6-28.2)*	(0.515)
All TBI	18.0 (14.2-27.3)		18.5 (13.2-31.4)		21.7 (15.6-36.9)	
OC (Ref:All TBI)	17.6 (12.9-23.0)	0.244	13.8 (10.8-17.8)	0.017	20.8 (16.8-24.8)**	(0.769)
HC (Ref:All TBI)	12.1 (10.3-14.3)	<0.001	12.6 (11.1-15.4)	<0.001	13.7 (11.3-21.7)	0.069
**7-12h**						
TBI/CT+	33.2 (22.8-51.8)		34.7 (19.6-59.0)		20.8 (13.5-28.0)	
TBI/CT- (Ref:TBI/CT+)	23.3 (15.4-36.9)	<0.001	17.7 (12.5-39.1)	<0.001	18.4 (10.7-20.9)*	0.192
All TBI	26.2 (16.4-43.8)		23.8 (15.1-49.6)		18.6 (13.4-25.2)	
OC (Ref:All TBI)	15.9 (11.4-27.7)*	0.009	19.5 (12.8-31.4)*	0.306	20.0 (15.2-27.4)**	(0.597)
HC (Ref:All TBI)	12.1 (10.3-14.3)	<0.001	12.6 (11.1-15.4)	<0.001	13.7 (11.3-21.7)	0.222
**13-24h**						
TBI/CT+	30.2 (19.7-48.9)		23.0 (14.8-43.4)		18.0 (13.3-31.8)	
TBI/CT- (Ref:TBI/CT+)	18.3 (13.3-29.1)	<0.001	17.9 (13.2-24.9)	<0.001	18.7 (15.3-32.1)	0.905
All TBI	22.9 (15.2-40.0)		19.5 (14.1-34.2)		18.2 (14.1-31.9)	
OC (Ref:All TBI)	15.1 (12.1-21.2)	<0.001	15.6 (12.0-19.9)	0.001	13.3 (9.7-17.1)**	(0.010)
HC (Ref:All TBI)	12.1 (10.3-14.3)	<0.001	12.6 (11.1-15.4)	<0.001	13.7 (11.3-21.7)	0.049

Notes:
*N=10 to <20 in this sub-group
**N<10 in this sub-group.
P-values are from the Wilcoxon Rank Sum test comparing the biomarkers between diagnostic groups.
P-values for comparisons in which at least one cell contains fewer than 10 individuals are shown in parentheses.
Abbreviations: GFAP = glial fibrillary acidic protein; Ref = reference group; TBI = traumatic brain injury; OC = orthopedic trauma control; HC = healthy control; UCH-L1 = ubiquitin carboxy-terminal hydrolase L1; S100B = S100 calcium binding protein B; NSE = neuron specific enolase

Supplemental Table 5. Discriminative value of day 1 blood-based biomarkers stratified by age and sampling time interval

Values are AUC (95% CI)	**17-39y**	**40-64y**	**65-90y**
**GFAP**			
TBI:CT+ vs. TBI:CT-			
0-6h	**0.906 (0.834,0.978)**	**0.867 (0.796,0.938)**	**0.895 (0.775,1)**
7-12h	**0.850 (0.802,0.898)**	**0.845 (0.781,0.91)**	0.775 (0.613,0.936)
13-24h	**0.868 (0.842,0.893)**	**0.841 (0.807,0.875)**	**0.828 (0.760,0.896)**
TBI vs. OC			
0-6h	**0.864 (0.812,0.916)**	**0.825 (0.749,0.902)**	0.685 (0.130,1)
7-12h	**0.900 (0.844,0.956)**	**0.910 (0.833,0.987)**	**0.922 (0.827,1)**
13-24h	**0.901 (0.872,0.929)***	**0.934 (0.904,0.963)***	**0.978 (0.956,0.999)**
TBI vs. HC			
0-6h	**0.899 (0.865,0.933)**	**0.878 (0.830,0.926)**	**0.912 (0.825,1)**
7-12h	**0.976 (0.962,0.989)**	**0.965 (0.940,0.990)**	**0.942 (0.885,1)**
13-24h	**0.959 (0.948,0.971)**	**0.969 (0.956,0.982)**	**0.972 (0.950,0.994)**
**UCH-L1**			
TBI:CT+ vs. TBI:CT-			
0-6h	**0.876 (0.798,0.953)**	0.700 (0.591,0.808)	0.678 (0.408,0.948)
7-12h	0.773 (0.712,0.834)	0.739 (0.653,0.825)	0.604 (0.425,0.784)
13-24h	0.752 (0.717,0.787)*	0.693 (0.647,0.738)	0.589 (0.487,0.690)
TBI vs. OC			
0-6h	0.762 (0.688,0.835)	0.754 (0.663,0.846)	0.741 (0.224,1)
7-12h	0.605 (0.509,0.700)	0.696 (0.559,0.833)	0.620 (0.326,0.913)
13-24h	0.660 (0.606,0.713)	0.675 (0.605,0.745)	0.715 (0.564,0.865)
TBI vs. HC			
0-6h	**0.930 (0.899,0.961)**	**0.917 (0.878,0.956)**	**0.879 (0.786,0.972)**
7-12h	**0.923 (0.898,0.949)**	**0.895 (0.851,0.939)**	**0.873 (0.782,0.963)**
13-24h	**0.863 (0.835,0.892)**	**0.841 (0.798,0.884)**	0.772 (0.678,0.867)
**S100B**			
TBI:CT+ vs. TBI:CT-			
0-6h	**0.813 (0.720,0.906)**	0.772 (0.673,0.872)	0.599 (0.341,0.856)
7-12h	0.749 (0.683,0.815)	0.707 (0.616,0.798)	0.687 (0.513,0.860)
13-24h	0.703 (0.665,0.742)	0.710 (0.664,0.756)	0.623 (0.53,0.717)
TBI vs. OC			
0-6h	0.636 (0.551,0.720)	0.755 (0.669,0.841)	0.648 (0.092,1)
7-12h	0.633 (0.514,0.752)	0.690 (0.555,0.826)	0.559 (0.307,0.811)
13-24h	0.671 (0.611,0.731)	0.687 (0.619,0.755)	0.675 (0.553,0.798)
TBI vs. HC			
0-6h	**0.901 (0.865,0.937)**	**0.889 (0.842,0.936)**	**0.896 (0.812,0.979)**
7-12h	**0.946 (0.921,0.971)**	**0.909 (0.865,0.953)**	**0.885 (0.797,0.972)**
13-24h	**0.888 (0.806,0.916)**	**0.842 (0.793,0.891)**	**0.826 (0.736,0.917)**
**NSE**			
TBI:CT+ vs. TBI:CT-			
0-6h	0.743 (0.626,0.859)	0.634 (0.520,0.747)	0.586 (0.309,0.862)
7-12h	0.650 (0.577,0.723)	0.676 (0.580,0.772)	0.637 (0.438,0.836)
13-24h	0.678 (0.638,0.717)*	0.611 (0.560,0.661)	0.507 (0.405,0.608)
TBI vs. OC			
0-6h	0.560 (0.461,0.659)	0.661 (0.556,0.766)	0.574 (0.015,1)
7-12h	0.680 (0.544,0.815)	0.587 (0.413,0.760)	0.578 (0.296,0.861)
13-24h	0.668 (0.603,0.733)	0.639 (0.565,0.713)	0.756 (0.628,0.885)
TBI vs. HC			
0-6h	0.764 (0.706,0.821)	0.729 (0.656,0.802)	0.653 (0.489,0.818)
7-12h	**0.836 (0.791,0.880)***	**0.801 (0.739,0.863)***	0.596 (0.438,0.755)
13-24h	**0.806 (0.767,0.846)***	0.770 (0.719,0.821)	0.630 (0.497,0.762)

Notes: **Bolded result** indicates AUC at least 0.8 (at least "good" discrimination).
*P<0.05 vs. 65-90y
Abbreviations: AUC = area under the curve; CI = confidence interval. All other abbreviations as defined in Supplemental Table 4.

Supplemental Table 6. Characteristics of Longitudinal TBI Cohort

**Characteristic**<br>N(%) or mean±standard deviation	**17-39y**<br>**N=261**	**40-64y**<br>**N=198**	**65-90**<br>**N=63**
**Age, years**	27.2±6.4	52.2±7.1	73.3±6.4
**Female**	79 (30.27%)	37 (18.69%)	25 (39.68%)
**Race**			
White	196 (75.68%)	172 (87.31%)	57 (90.48%)
Black	42 (16.22%)	18 (9.14%)	3 (4.76%)
Other	21 (8.11%)	7 (3.55%)	3 (4.76%)
**Hispanic**	53 (20.46%)	49 (24.87%)	7 (11.11%)
**Education, years**	12.8±2.2	13.0±3.4	14.2±3.7
**Past medical history**			
Hypertension	9 (3.45%)	54 (27.27%)	29 (46.03%)
Hyperlipidemia	3 (1.15%)	17 (8.59%)	16 (25.4%)
Ischemic heart disease	0 (0%)	0 (0%)	3 (4.76%)
Stroke or transient ischemic attack	1 (0.38%)	2 (1.01%)	5 (7.94%)
Diabetes	3 (1.15%)	27 (13.64%)	10 (15.87%)
Renal disease	9 (3.45%)	9 (4.55%)	10 (15.87%)
Pulmonary disease	17 (6.51%)	22 (11.11%)	13 (20.63%)
Prior traumatic brain injury	61 (24.02%)	47 (24.48%)	11 (17.74%)
Psychiatric history	56 (21.46%)	45 (22.73%)	15 (23.81%)
**Presenting Glasgow Coma Scale**			
3-8	90 (35.43%)	51 (26.7%)	4 (6.67%)
9-12	22 (8.66%)	23 (12.04%)	3 (5%)
13-15	142 (55.91%)	117 (61.26%)	53 (88.33%)
**Disposition**			
Emergency department discharge	0 (0%)	0 (0%)	0 (0%)
Hospital ward admit	64 (24.52%)	40 (20.2%)	21 (33.33%)
Intensive care unit admit	197 (75.48%)	158 (79.8%)	42 (66.67%)

Supplemental Table 7. Blood-Based Biomarker Evolution over Two Weeks by Age

Values are median (interquartile range)	17-39y	N	P	40-64y	N	P	65-90y	N	P
**GFAP, pg/mL**									
**Day 1**									
TBI/CT+	2607.7 (1218.2-6229.1)	168		2123.7(1058.8-4131.6)	134		2079.0(633.6-4192.0)	49	
TBI/CT- (Ref:TBI/CT+)	277.2(94.2-820.4)	87	<0.001	374.6(88.7-1045.4)	54	<0.001	157.0(95.0-301.3)	13	<0.001
All TBI	1481.7 (450.2-4272.7)	261		1452.4(496.5-3085.5)	198		1054.7(316.6-3433.9)	63	
OC (Ref:All TBI)	17.6 (9.3-40.8)	10	<0.001	14.7(9.3-17.6)	10	<0.001	19.3(14.0-126.2)	4	(0.007)
HC (Ref: All TBI)	5.0 (2.0-10.0)	129	<0.001	10.0(5.0-15.0)	58	<0.001	28.5(25.3-38.8)	22	<0.001
**Day 3**									
TBI/CT+	650.8(238.3-1764.3)	158		781.3(197.6-2898.0)	128		602.4(270.8-1752.3)	46	
TBI/CT- (Ref:TBI/CT+)	29.4(10.6-70.6)	80	<0.001	55.0(15.2-119.1)	51	<0.001	94.1(37.0-128.5)	13	<0.001
All TBI	257.9(48.9-1179.0)	243		332.0(81.5-1610.8)	188		408.9(119.1-1291.7)	60	
OC (Ref:All TBI)	12.9(6.2-19.1)	7	(<0.001)	6.4(2.5-7.5)	9	(<0.001)	17.8(16.3-19.2)	2	(0.024)
HC (Ref: All TBI)	5.0 (2.0-10.0)	129	<0.001	10.0(5.0-15.0)	58	<0.001	28.5(25.3-38.8)	22	<0.001
**Day 5**									
TBI/CT+	218.6(100.3-639.6)	114		276.18(70.0-1081.6)	92		286.4(106.4-1216.6)	29	
TBI/CT- (Ref:TBI/CT+)	15.2(9.8-41.8)	39	<0.001	19.3(12.6-30.1)	28	<0.001	36.0(36.0-36.0)	1	(N/A)
All TBI	136.3(35.0-451.9)	157		157.0(27.7-873.8)	127		212.3(96.0-1038.3)	31	
OC (Ref:All TBI)	4.5(3.4-5.0)	4	(0.001)	1.9(1.6-4.0)	4	(<0.001)	22.5(19.7-22.9)	4	(0.004)
HC (Ref: All TBI)	5.0 (2.0-10.0)	129	<0.001	10.0(5.0-15.0)	58	<0.001	28.5(25.3-38.8)	22	<0.001
**Week 2**									
TBI/CT+	64.4(36.3-144.4)	168		46.0(22.0-131.0)	134		59.9(33.9-102.3)	49	
TBI/CT- (Ref:TBI/CT+)	13.4(7.4-24.9)	87	<0.001	17.5(12.0-26.3)	54	<0.001	29.0(20.5-41.8)	13	0.0187
All TBI	24.9(15.8-105.5)	261		34.4(16.6-99.5)	198		54.2(26.9-96.8)	63	
OC (Ref:All TBI)	7.0(4.2-10.6)	10	<0.001	6.0(2.9-8.3)	10	<0.001	21.2(16.3-24.0)	4	(0.0129)
HC (Ref: All TBI)	5.0 (2.0-10.0)	129	<0.001	10.0(5.0-15.0)	58	<0.001	28.5(25.3-38.8)	22	0.009
**UCH-L1, pg/mL**									
**Day 1**									
TBI/CT+	458.6(263.1- 1110.9)	168		504.1(249.6-1012.5)	134		276.0(164.0-666.8)	49	
TBI/CT- (Ref:TBI/CT+)	280.8(155.6- 548.8)	87	<0.001	271.1(173.7-441.7)	54	<0.001	224.0(142.5-265.2)	13	0.265
All TBI	409.5(203.6- 890.0)	261		397.1(199.1-888.3)	198		256.8(158.1-639.6)	63	
OC (Ref:All TBI)	150.3(90.5- 240.4)	10	0.001	165.4(116.4-211.4)	10	0.005	187.1(88.0-311.4)	4	(0.261)
HC (Ref:All TBI)	47.0(37.0- 68.0)	129	<0.001	74.0(52.3-96.0)	58	<0.001	109.0(75.3-156.8)	22	<0.001
**Day 3**									
TBI/CT+	101.9(56.0- 172.7)	158		117.3(66.6-208.6)	128		112.7(67.4-167.2)	46	
TBI/CT- (Ref:TBI/CT+)	77.1(50.7- 128.5)	80	0.027	75.0(55.0-128.6)	51	0.003	106.3(71.0-143.5)	13	0.766
All TBI	91.9(54.0- 146.3)	243		109.6(63.3-192.2)	188		112.7(69.3-165.6)	60	
OC (Ref:All TBI)	83.9(66.1- 115.7)	7	(0.981)	54.9(54.2-87.4)	9	(0.009)	170.8(150.9-190.6)	2	(0.329)
HC (Ref:All TBI)	47.0(37.0- 68.0)	129	<0.001	74.0(52.3-96.0)	58	<0.001	109.0(75.3-156.8)	22	0.754
**Day 5**									
TBI/CT+	80.2(50.3-128.1)	114		107.0(78.6-159.7)	92		105.6(75.406-149.062)	29	
TBI/CT- (Ref:TBI/CT+)	73.8(53.5-120.8)	39	0.680	84.7(63.6-129.2)	28	0.095	50.0(50.0-50.0)	1	(N/A)
All TBI	80.2(52.7-125.5)	157		105.2(75.5-152.9)	127		105.6(73.9- 142.5)	31	

OC (Ref:All TBI)	73.4(57.4-84.9)	4	(0.441)	74.7(59.6-103.1)	4	(0.276)	111.95(66.35-173.507)	4	(0.940)
HC (Ref:All TBI)	47.0(37.0- 68.0)	129	<0.001	74.0(52.3-96.0)	58	<0.001	109.0(75.3-156.8)	22	0.738
**Week 2**									
TBI/CT+	81.6(52.1-129.7)	168		106.0(70.5-179.2)	134		113.4(74.8-181.6)	49	
TBI/CT- (Ref:TBI/CT+)	68.9(42.9-93.7)	87	0.014	80.5(53.6-117.8)	54	0.006	109.0(81.4-130.8)	13	0.505
All TBI	76.5(49.1-125.1)	261		102.6(64.7-168.0)	198		109.3(76.4-171.3)	63	
OC (Ref:All TBI)	71.2(61.8-91.4)	10	0.886	92.2(57.6-104.7)	10	0.237	100.5(56.0-147.8)	4	(0.615)
HC (Ref:All TBI)	47.0(37.0- 68.0)	129	<0.001	74.0(52.3-96.0)	58	<0.001	109.0(75.3-156.8)	22	0.794
**S100B**									
**Day 1**									
TBI/CT+	0.286(0.144-0.560)	163		0.229(0.140-0.600)	134		0.206(0.112-0.399)	47	
TBI/CT- (Ref:TBI/CT+)	0.192(0.090-0.301)	82	<0.001	0.145(0.074-0.251)	53	<0.001	0.096(0.083-0.155)	13	0.041
All TBI	0.243(0.134-0.466)	251		0.204(0.119-0.416)	197		0.164(0.096-0.360)	61	
OC (Ref:All TBI)	0.0690(0.064-0.092)	10	<0.001	0.096(0.081-0.132)	10	0.003	0.071(0.062-0.082)	4	(0.011)
HC (Ref:All TBI)	0.041(0.032-0.056)	129	<0.001	0.051(0.035-0.066)	58	<0.001	0.043(0.035-0.074)	22	<0.001
**Day 3**									
TBI/CT+	0.096(0.056-0.178)	151		0.106(0.062-0.208)	128		0.110(0.069-0.154)	44	
TBI/CT- (Ref:TBI/CT+)	0.077(0.052-0.104)	75	0.015	0.065(0.044-0.084)	49	<0.001	0.055(0.047-0.084)	13	0.011
All TBI	0.086(0.054-0.148)	231		0.092(0.054-0.177)	185		0.084(0.062-0.144)	58	
OC (Ref:All TBI)	0.043(0.039-0.072)	7	(0.032)	0.047(0.043-0.070)	9	(0.036)	0.062(0.054-0.071)	2	(0.294)
HC (Ref:All TBI)	0.041(0.032-0.056)	129	<0.001	0.051(0.035-0.066)	58	<0.001	0.043(0.035-0.074)	22	<0.001
**Day 5**									
TBI/CT+	0.064(0.044-0.101)	110		0.086(0.051-0.130)	91		0.116(0.077-0.173)	29	
TBI/CT- (Ref:TBI/CT+)	0.074(0.041-0.105)	36	0.987	0.054(0.039-0.073)	26	0.003	0.071(0.071-0.071)	1	(N/A)
All TBI	0.066(0.043-0.103)	150		0.074(0.046-0.120)	124		0.115(0.077-0.159)	31	
OC (Ref:All TBI)	0.063(0.054-0.072)	4	(0.760)	0.049(0.045-0.051)	4	(0.095)	0.068(0.061-0.090)	4	(0.161)
HC (Ref:All TBI)	0.041(0.032-0.056)	129	<0.001	0.051(0.035-0.066)	58	<0.001	0.043(0.035-0.074)	22	<0.001
**Week 2**									
TBI/CT+	0.05(0.031-0.073)	129		0.048(0.032-0.076)	102		0.063(0.048-0.100)	41	
TBI/CT- (Ref:TBI/CT+)	0.051(0.029-0.085)	45	0.973	0.044(0.034-0.056)	29	0.350	0.053(0.044-0.064)	8	(0.256)
All TBI	0.050(0.030-0.073)	180		0.047(0.033-0.074)	141		0.062(0.047-0.088)	50	
OC (Ref:All TBI)	0.039(0.034-0.050)	9	(0.173)	0.052(0.038-0.077)	10	0.613	0.038(0.033-0.059)	4	(0.306)
HC (Ref:All TBI)	0.041(0.032-0.056)	129	0.033	0.051(0.035-0.066)	58	0.970	0.043(0.035-0.074)	22	0.171
**NSE**									
**Day 1**									
TBI/CT+	34.48(23.12-53.63)	163		26.30(19.19-50.47)	134		19.55(13.75-37.7)	47	
TBI/CT- (Ref:TBI/CT+)	24.23(14.96-42.73)	82	<0.001	20.47(15.26-29.79)	53	0.007	18.45(16.06-37.72)	13	>0.999
All TBI	31.64(20.34-49.61)	251		24.33(18.24-47.59)	197		19.27(14.97-37.72)	61	
OC (Ref:All TBI)	14.16(13.00-15.51)	10	<0.001	13.02(9.663-21.59)	10	0.016	13.02(8.613-27.14)	4	(0.346)
HC (Ref:All TBI)	12.13(10.27-14.28)	129	<0.001	12.59(11.12-15.45)	58	<0.001	13.72(11.34-21.74)	22	0.049
**Day 3**									
TBI/CT+	15.73(10.91-24.07)	151		15.50(11.72-22.52)	128		13.16(10.03-19.88)	44	
TBI/CT- (Ref:TBI/CT+)	13.03(10.62-16.60)	75	0.014	12.80(9.96-19.80)	49	0.104	13.34(12.89-15.57)	13	0.939
All TBI	14.59(10.66-20.77)	231		14.85(11.10-22.06)	186		13.16(10.58-17.41)	58	
OC (Ref:All TBI)	9.85(7.96-26.39)	7	(0.547)	8.61(6.86-13.02)	9	(0.004)	17.29(15.21-19.36)	2	(0.422)

HC (Ref:All TBI)	12.13(10.27-14.28)	129	<0.001	12.59(11.12-15.45)	58	0.009	13.72(11.34-21.74)	22	0.316
**Day 5**									
TBI/CT+	14.53(10.62-22.61)	110		15.15(12.04-24.055)	91		14.93(10.63-18.58)	29	
TBI/CT- (Ref:TBI/CT+)	15.02(11.80-19.60)	36	0.910	12.81(11.16-15.33)	26	0.100	20.88(20.88-20.88)	1	(N/A)
All TBI	14.50(10.67-20.54)	150		14.39(11.50-22.84)	124		14.93(10.58-19.73)	31	
OC (Ref:All TBI)	15.79(14.00-17.19)	4	(0.977)	10.06(9.00-10.71)	4	(0.024)	17.36(12.39-23.09)	4	(0.531)
HC (Ref:All TBI)	12.13(10.27-14.28)	129	0.001	12.59(11.12-15.45)	58	0.012	13.72(11.34-21.74)	22	0.549
**Week 2**									
TBI/CT+	19.41(13.56-27.19)	129		18.7(14.38-33.82)	103		16.18(13.14-21.91)	41	
TBI/CT- (Ref:TBI/CT+)	14.41(10.38-18.47)	45	<0.001	14.09(11.63-20.28)	29	0.006	15.70(12.41-21.16)	8	(0.760)
All TBI	17.41(12.86-25.50)	180		17.34(13.6-28.21)	142		16.05(12.87-21.89)	50	
OC (Ref:All TBI)	16.46(13.59-17.46)	9	(0.820)	12.79(9.74-14.14)	10	0.005	14.38(13.76-16.89)	4	(0.804)
HC (Ref:All TBI)	12.13(10.27-14.28)	129	<0.001	12.59(11.12-15.45)	58	<0.001	13.72(11.34-21.74)	22	0.467

Notes: HC samples were drawn only at baseline as there is no index injury for comparison. Baseline HC biomarker levels are compared to longitudinal levels in patients with TBI. P-values for comparisons in which at least one cell contains fewer than 10 individuals are shown in parentheses.
Abbreviations: As defined in Supplemental Table 4.

Supplemental Table 8. Discriminative value of day 1, day 3, day 5, and week 2 UCH-L1, NSE, and S100B stratified by age

Values are AUC (95% CI)	**17-39y**	**40-64y**	**65-90y**
**UCHL-1**			
**TBI/CT+ vs. TBI/CT-**			
Day 1	0.656 (0.586,0.725)	0.656 (0.571,0.742)	0.603 (0.434,0.772)
Day 3	0.588 (0.513,0.662)	0.645 (0.556,0.733)	0.528 (0.350,0.707)
Day 5	0.522 (0.421,0.623)	0.605 (0.479,0.730)	NA**
Week 2	0.594 (0.521,0.667)	0.629 (0.541,0.717)	0.562 (0.398,0.726)
**TBI vs. OC**			
Day 1	0.799 (0.701,0.897)	0.766 (0.627,0.904)	0.671 (0.358,0.983)
Day 3	0.497 (0.305,0.689)	0.758 (0.605,0.911)	0.708 (0.474,0.943)
Day 5	0.614 (0.400,0.828)	0.661 (0.370,0.953)	0.484 (0.098,0.870)
Week 2	0.486 (0.337,0.636)	0.611 (0.468,0.755)	0.577 (0.192,0.963)
**TBI vs. HC**			
Day 1	**0.963 (0.946,0.98)**	**0.932 (0.902,0.962)**	**0.800 (0.702,0.898)**
Day 3	0.751 (0.701,0.801)	0.675 (0.603,0.748)	0.477 (0.344,0.610)
Day 5	0.732 (0.674,0.790)	0.693 (0.612,0.774)	0.528 (0.369,0.687)
Week 2	0.703 (0.650,0.756)	0.651 (0.577,0.724)	0.481 (0.343,0.619)
**S100B**			
**TBI/CT+ vs. TBI/CT-**			
Day 1	0.637 (0.565,0.709)	0.696 (0.612,0.780)	0.687 (0.523,0.852)
Day 3	0.600 (0.523,0.676)	0.703 (0.622,0.784)	0.734 (0.570,0.898)
Day 5	0.499 (0.389,0.609)	0.691 (0.577,0.805)	NA**
Week 2	0.498 (0.400,0.596)	0.557 (0.444,0.607)	0.630 (0.421,0.838)
**TBI vs. OC**			
Day 1	**0.854 (0.764,0.944)**	0.777 (0.652,0.902)	**0.883 (0.783,0.984)**
Day 3	0.739 (0.573,0.905)	0.707 (0.561,0.853)	0.724 (0.407,1)
Day 5	0.563 (0.293,0.834)	0.747 (0.644,0.850)	0.722 (0.449,0.994)
Week 2	0.635 (0.506,0.763)	0.548 (0.379,0.717)	0.657 (0.325,0.990)
**TBI vs. HC**			
Day 1	**0.950 (0.925,0.974)**	**0.922 (0.887,0.957)**	**0.899 (0.825,0.972)**
Day 3	**0.821 (0.778,0.864)**	0.756 (0.690,0.821)	0.754 (0.635,0.874)
Day 5	0.736 (0.678,0.794)	0.691 (0.612,0.771)	**0.818 (0.698,0.938)**
Week 2	0.571 (0.508,0.635)	0.502 (0.416,0.588)	0.602 (0.452,0.753)
**NSE**			
**TBI/CT+ vs. TBI/CT-**			
Day 1	0.631 (0.555,0.708)	0.628 (0.542,0.714)	0.499 (0.331,0.667)
Day 3	0.601 (0.526,0.676)	0.579 (0.481,0.677)	0.508 (0.349,0.667)
Day 5	0.494 (0.391,0.596)	0.606 (0.486,0.727)	NA**
Week 2	0.694 (0.606,0.782)	0.669 (0.559,0.778)	0.537 (0.286,0.787)
**TBI vs. OC**			
Day 1	**0.841 (0.742,0.939)**	0.726 (0.529,0.923)	0.643 (0.231,1)
Day 3	0.567 (0.258,0.876)	0.789 (0.650,0.929)	0.672 (0.321,1)
Day 5	0.505 (0.298,0.712)	**0.833 (0.725,0.940)**	0.605 (0.339,0.871)
Week 2	0.523 (0.351,0.695)	0.765 (0.677,0.854)	0.540 (0.301,0.779)
**TBI vs. HC**			
Day 1	**0.871 (0.831,0.912)**	**0.851 (0.803,0.899)**	0.643 (0.501,0.784)
Day 3	0.608 (0.550,0.667)	0.614 (0.542,0.686)	0.573 (0.434,0.713)
Day 5	0.611 (0.544,0.679)	0.616 (0.535,0.698)	0.450 (0.292,0.609)
Week 2	0.715 (0.656,0.773)	0.717 (0.646,0.788)	0.555 (0.402,0.707)

Notes: **Bolded result** indicates AUC at least 0.8 (at least “good” discrimination).
****N=1 in the TBI CT- group**
Abbreviations: As defined in Supplemental Tables 4 and 5.

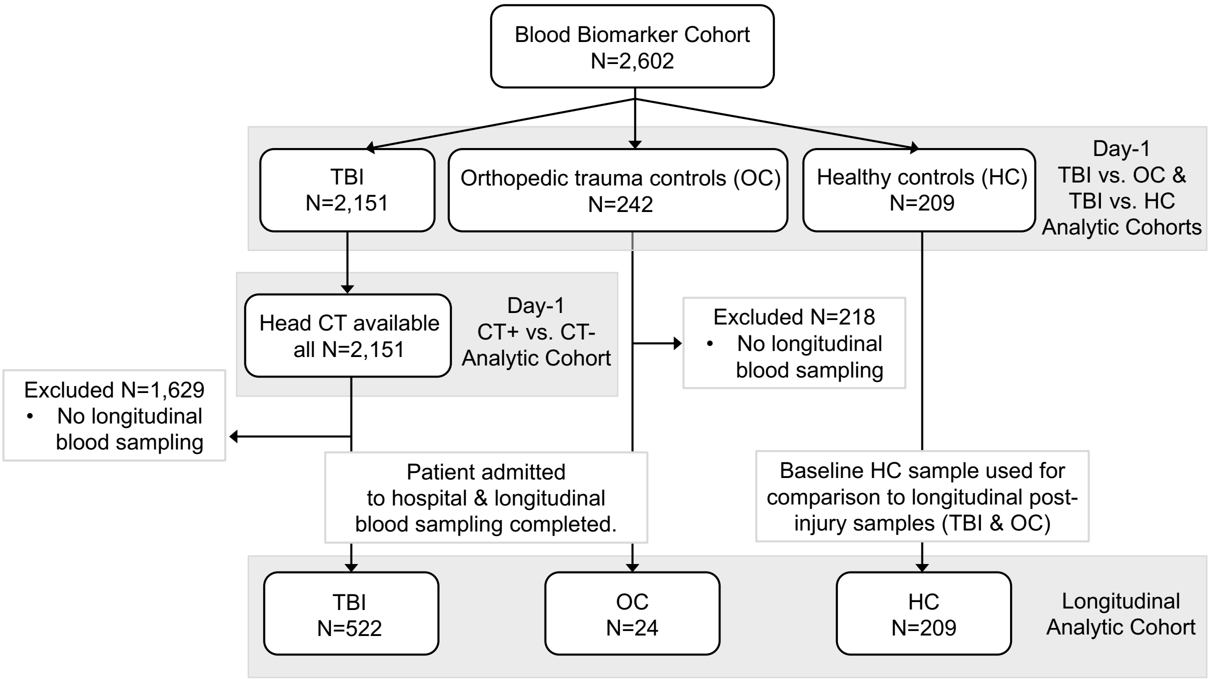

Supplemental Figure 1. Selection of analytic cohorts

Participant flow diagram showing selection of cohorts for the day-1 and longitudinal analyses. Abbreviations: TBI = traumatic brain injury

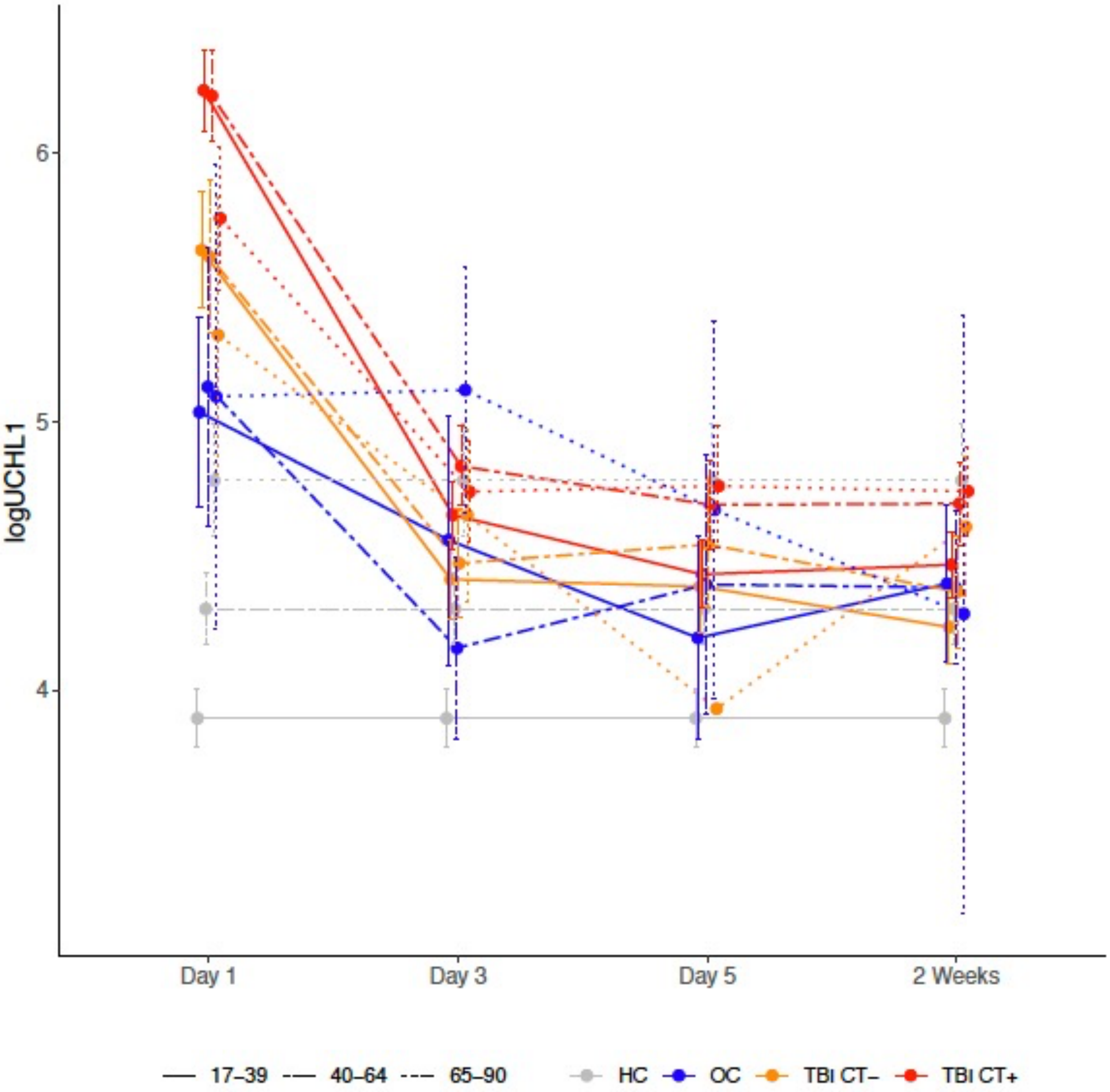

Supplemental Figure 2. Longitudinal (Day 1 through Week 2) Blood-based UCHL1 Levels Stratified by Diagnostic Group and Age
Mean with 95% confidence intervals is shown for log-transformed UCHL1 levels stratified by age category and diagnostic group, with the same color coding as in Figures 3 and 4. Healthy control biomarker levels were only drawn at a single time-point so the same values are plotted here repeatedly for comparison to the other diagnostic groups over time. Specific blood-biomarker levels and results of Wilcoxon Rank Sum tests comparing levels across diagnostic groups, stratified by age and days post-injury, are reported in Supplemental Table 7.

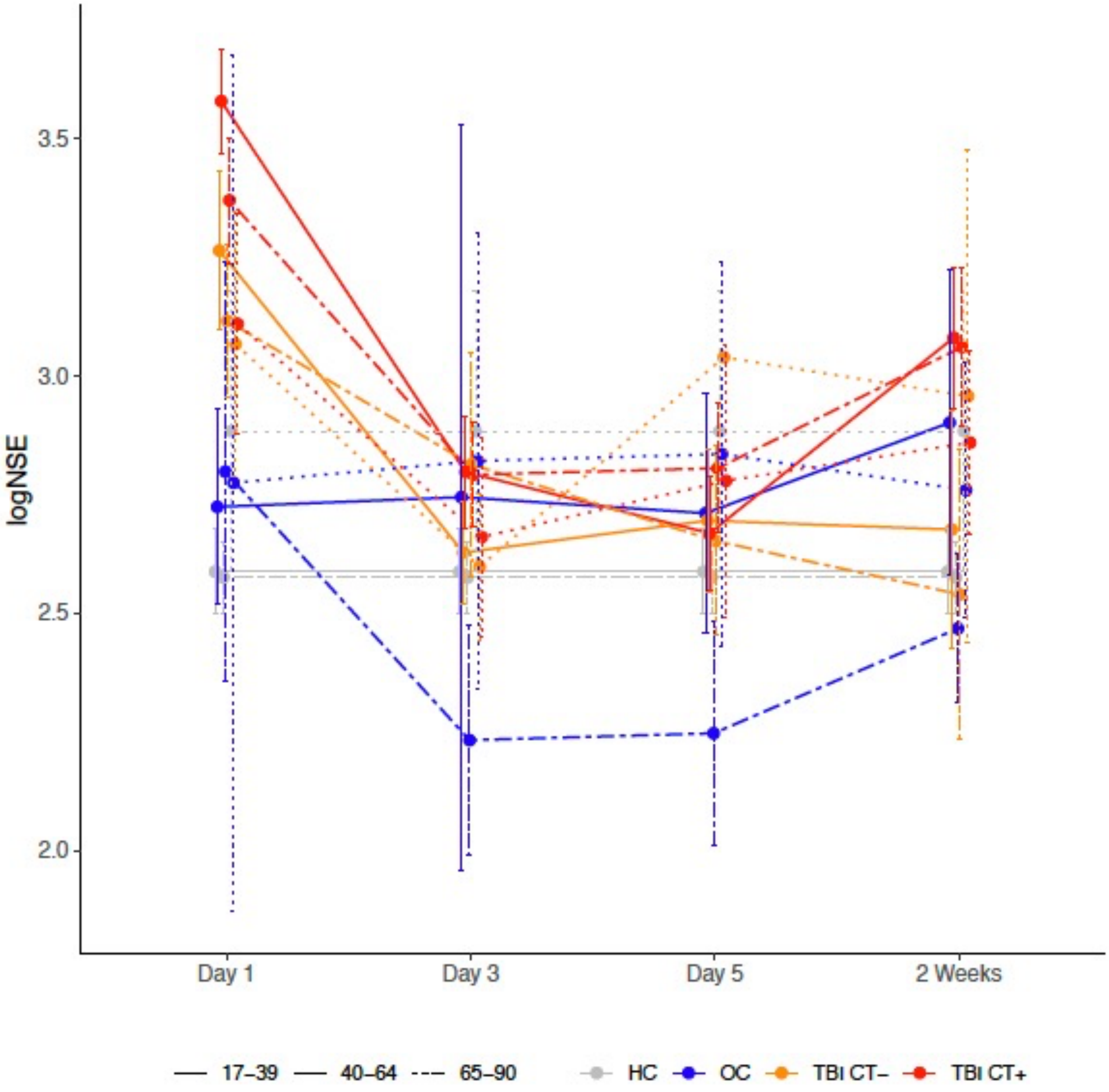

Supplemental Figure 3. Longitudinal (Day 1 through Week 2) Blood-based NSE Levels Stratified by Diagnostic Group and Age
Mean with 95% confidence intervals is shown for log-transformed NSE levels stratified by age category and diagnostic group, with the same color coding as in Figures 3 and 4. Healthy control biomarker levels were only drawn at a single time-point so the same values are plotted here repeatedly for comparison to the other diagnostic groups over time. Specific blood-biomarker levels and results of Wilcoxon Rank Sum tests comparing levels across diagnostic groups, stratified by age and days post-injury, are reported in Supplemental Table 7.

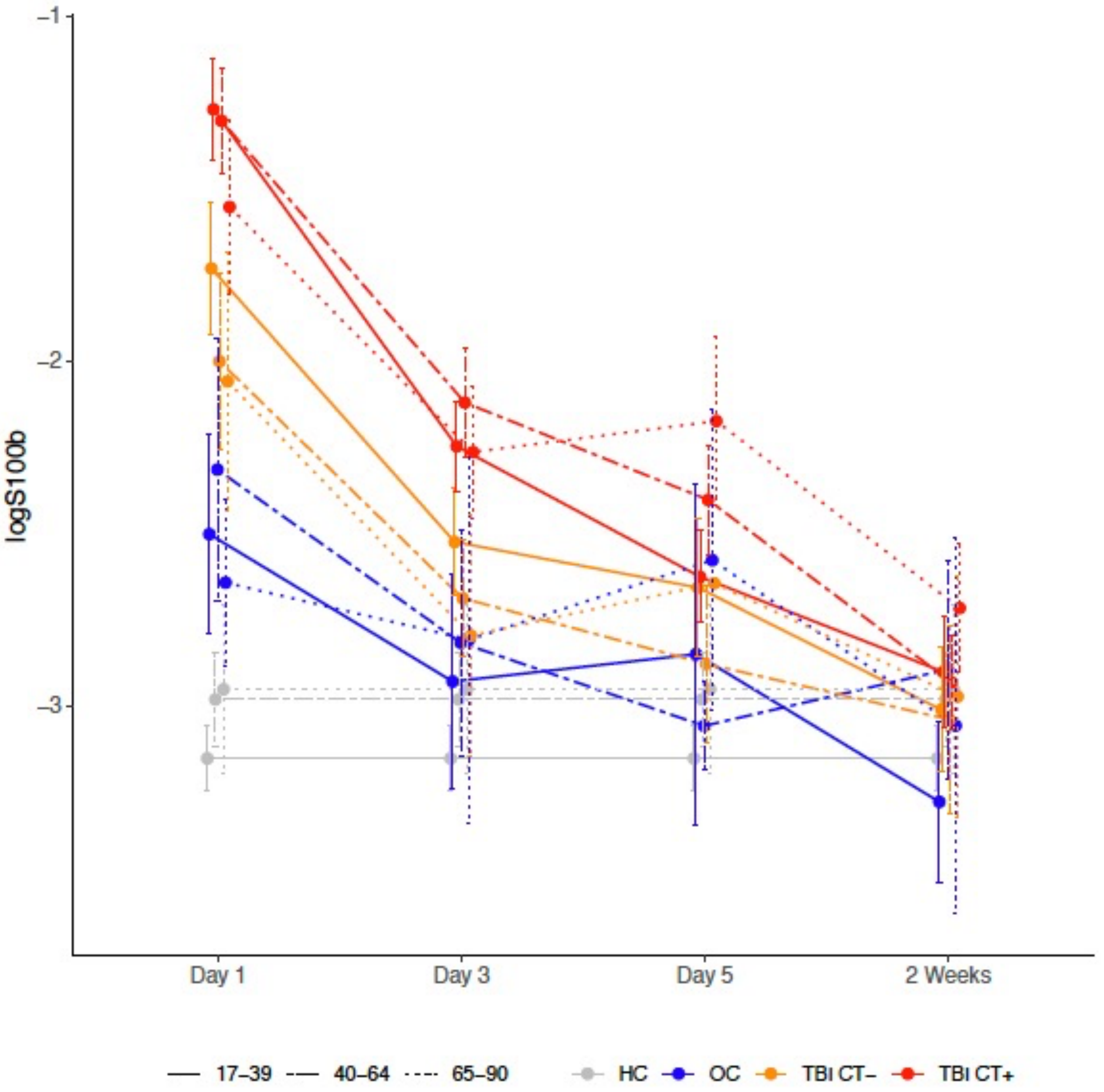

Supplemental Figure 4. Longitudinal (Day 1 through Week 2) Blood-based S100b Levels Stratified by Diagnostic Group and Age
Mean with 95% confidence intervals is shown for log-transformed S100b levels stratified by age category and diagnostic group, with the same color coding as in Figures 3 and 4. Healthy control biomarker levels were only drawn at a single time-point so the same values are plotted here repeatedly for comparison to the other diagnostic groups over time. Specific blood-biomarker levels and results of Wilcoxon Rank Sum tests comparing levels across diagnostic groups, stratified by age and days post-injury, are reported in Supplemental Table 7.
